# Extemporaneous Compounding, Pharmacy Preparations and Related Product Care in the Netherlands

**DOI:** 10.3390/pharmaceutics17081005

**Published:** 2025-07-31

**Authors:** Herman J. Woerdenbag, Boy van Basten, Christien Oussoren, Oscar S. N. M. Smeets, Astrid Annaciri-Donkers, Mirjam Crul, J. Marina Maurer, Kirsten J. M. Schimmel, E. Marleen Kemper, Marjolijn N. Lub-de Hooge, Nanno Schreuder, Melissa Eikmann, Arwin S. Ramcharan, Richard B. Lantink, Julian Quodbach, Hendrikus H. Boersma, Oscar Kelder, Karin H. M. Larmené-Beld, Paul P. H. Le Brun, Robbert Jan Kok, Reinout C. A. Schellekens, Oscar Breukels, Henderik W. Frijlink, Bahez Gareb

**Affiliations:** 1 Department of Pharmaceutical Technology and Biopharmacy, Groningen Research Institute of Pharmacy (GRIP), University of Groningen, Antonius Deusinglaan 1, 9713 AV Groningen, The Netherlands; h.w.frijlink@rug.nl; 2 Department of Pharmaceutical Technology and Biopharmacy, School of Science and Engineering (SSE), University of Groningen, Antonius Deusinglaan 1, 9713 AV Groningen, The Netherlands; b.van.basten@rug.nl; 3 Royal Dutch Pharmacists Association (KNMP), Alexanderstraat 11, 2514 JL Den Haag, The Netherlands; c.oussoren@knmp.nl (C.O.); o.s.n.m.smeets@knmp.nl (O.S.N.M.S.); a.annaciri-donkers@knmp.nl (A.A.-D.); 4 Department of Clinical Pharmacology and Pharmacy, Amsterdam University Medical Center, Location Vrije Universiteit, De Boelelaan 1117, 1081 HV Amsterdam, The Netherlands; m.crul@amsterdamumc.nl; 5 Department of Clinical Pharmacy and Pharmacology, University Medical Center Groningen (UMCG), Hanzeplein 1, 9713 GZ Groningen, The Netherlands; m.maurer@umcg.nl (J.M.M.); m.n.de.hooge@umcg.nl (M.N.L.-d.H.); h.h.boersma@umcg.nl (H.H.B.); b.gareb01@umcg.nl (B.G.); 6 Department of Clinical Pharmacy and Toxicology, Leiden University Medical Center (LUMC), Albinusdreef 2, 2333 ZA Leiden, The Netherlands; k.j.m.schimmel@lumc.nl (K.J.M.S.); pphlebrun@icloud.com (P.P.H.L.B.); 7 Apotheek A15, Buys Ballotstraat 2, 4207 HT Gorinchem, The Netherlands; m.kemper@apotheeka15.nl; 8 GE HealthCare Radiopharmacy Zwolle, Dokter Spanjaardweg 1A, 8025 AC Zwolle, The Netherlands; nanno.schreuder@gehealthcare.com; 9 Transvaal Apotheek, Kempstraat 113, 2572 GC Den Haag, The Netherlands; m.eikmann@transvaalapotheek.nl (M.E.); a.ramcharan@transvaalapotheek.nl (A.S.R.); 10 Laboratorium Ofichem, Heembadweg 5, 9561 CZ Ter Apel, The Netherlands; lantink@ofichem.com; 11 Division of Pharmaceutics, Utrecht Institute for Pharmaceutical Sciences (UIPS), Utrecht University, Universiteitsweg 99, 3584 GC Utrecht, The Netherlands; j.h.j.quodbach@uu.nl (J.Q.); r.j.kok@uu.nl (R.J.K.); 12 Ziekenhuis Groep Twente (ZGT) Apotheek, Boerhaavelaan 63, 7555 BB Hengelo, The Netherlands; okelder@home.nl; 13 Department of Clinical Pharmacy, Isala Hospital, Dokter van Heesweg 2, 8025 AB Zwolle, The Netherlands; k.h.m.beld@isala.nl; 14 Fagron Sterile Services Nederland, Dieselstraat 3, 7903 AR Hoogeveen, The Netherlands; reinout.schellekens@fagron.nl; 15 Department of Hospital Pharmacy, Meander Medisch Centrum Amersfoort, Maatweg 3, 3813 TZ Amersfoort, The Netherlands; o.breukels@meandermc.nl

**Keywords:** advanced therapy medicinal products (ATMPs), biologicals, community pharmacy, extemporaneous compounding, hospital pharmacy, legislation and regulations, oncolytic medicinal products, personalised medicine, pharmaceutical product care, pharmacy education, pharmacy preparations, radiopharmaceuticals, total parenteral nutrition (TPN)

## Abstract

**Background/Objectives**: In many parts of the world, pharmacists hold the primary responsibility for providing safe and effective pharmacotherapy. A key aspect is the availability of appropriate medicines for each individual patient. When industrially manufactured medicines are unsuitable or unavailable, pharmacists can prepare tailor-made medicines. While this principle applies globally, practices vary between countries. In the Netherlands, the preparation of medicines in pharmacies is well-established and integrated into routine healthcare. This narrative review explores the role and significance of extemporaneous compounding, pharmacy preparations and related product care in the Netherlands. **Methods**: Pharmacists involved in pharmacy preparations across various professional sectors, including community and hospital pharmacies, central compounding facilities, academia, and the professional pharmacists’ organisation, provided detailed and expert insights based on the literature and policy documents while also sharing their critical perspectives. **Results**: We present arguments supporting the need for pharmacy preparations and examine their position and role in community and hospital pharmacies in the Netherlands. Additional topics are discussed, including the regulatory and legal framework, outsourcing, quality assurance, standardisation, education, and international context. Specific pharmacy preparation topics, often with a research component and a strong focus on product care, are highlighted, including paediatric dosage forms, swallowing difficulties and feeding tubes, hospital-at-home care, reconstitution of oncolytic drugs and biologicals, total parenteral nutrition (TPN), advanced therapy medicinal products (ATMPs), radiopharmaceuticals and optical tracers, clinical trial medication, robotisation in reconstitution, and patient-centric solid oral dosage forms. **Conclusions**: The widespread acceptance of pharmacy preparations in the Netherlands is the result of a unique combination of strict adherence to tailored regulations that ensure quality and safety, and patient-oriented flexibility in design, formulation, and production. This approach is further reinforced by the standardisation of a broad range of formulations and procedures across primary, secondary and tertiary care, as well as by continuous research-driven innovation to develop new medicines, formulations, and production methods.

## 1. Introduction and Aim

Pharmacists are an indispensable link in the healthcare chain, providing reliable pharmaceutical care to patients globally. Yet, while this core task is universal, specifics will differ from country to country, guided by national laws, regulations, customs and history. In the Netherlands, both community and hospital pharmacists legally share responsibility with other healthcare professionals for the pharmacotherapeutic treatment of patients. Dutch pharmacists are heavily involved in patient-centred tasks, such as counselling, instructing correct medication use, informing about possible adverse reactions, and supplying patients with the most appropriate medicine, either licensed or unlicensed [1].

Most prescribed and dispensed medicines are industrially manufactured and often mass-produced. These medicines are licensed, meaning that marketing authorisation has been granted following a registration procedure via a competent authority such as the European Medicines Agency (EMA) [2], or a national equivalent such as the Medicines Evaluation Board (MEB) (Dutch: College ter Beoordeling van Geneesmiddelen; CBG) in the Netherlands [3]. This procedure and the subsequent authorisation ensure the quality, effectiveness and safety of a pharmaceutical product. Although the vast majority of prescriptions in the Netherlands involve registered medicines nowadays [4,5], the market cannot always (sufficiently) provide the optimal medication for each specific patient or patient group. In such cases, pharmacists are allowed to either prepare (customised) medicines or outsource the preparation thereof to specialised central compounding facilities before dispensing them to the patient. This practice is known as extemporaneous compounding, yielding so-called pharmacy preparations [6].

Worldwide, extemporaneous compounding is acknowledged and defined as the practice in which a licensed pharmacist, or a person under the supervision of a licensed pharmacist, mixes or alters ingredients based on a prescription to create a pharmacy preparation tailored to the medical needs of an individual patient or patient group, in cases where treatment with a commercially available medicinal product is not possible [7,8,9,10]. In Europe, pharmacy preparations are considered indispensable for meeting the specific demands of individual patients [7,10]. They are an integral part of daily pharmacy practice in the Netherlands, though the extent varies depending on the pharmacy setting, and they fall under the umbrella of pharmaceutical product care.

In the Netherlands, the concept of pharmaceutical product care refers to ensuring that patients receive the most appropriate medicine upon prescription, in an optimal dosage form, and of the highest possible quality. It encompasses the procurement, storage, preparation (or outsourcing thereof, where applicable), and dispensing of medicines, along with providing patients with instructions on their use. Pharmaceutical product care is intrinsically linked to effective pharmacotherapeutic treatment and pharmaceutical patient care [11].

Non-industrially prepared medicines are unlicensed; they lack the rigorous governmental control of licensed products. Extemporaneous compounding, however, is closely regulated in the Netherlands. To ensure quality and safety, pharmacists must adhere to specific guidelines and rules that have been developed based on those for industrially manufactured products, such as Good Manufacturing Practice (GMP) [12,13,14], International Conference on Harmonisation (ICH) [15], and Pharmaceutical Inspection Convention/Pharmaceutical Inspection Co-operation Scheme (PIC/s) [16]. Furthermore, as a member state of the European Union (EU), all medicinal products should comply with the European Pharmacopoeia (Ph. Eur.) monograph ‘Pharmaceutical Preparations’ [6].

Knowledge of a product’s composition, structure, and functionality, as well as the production process and the implications of its properties for efficacy and safety, primarily falls within the domain of pharmacists. They are the only healthcare professionals who combine pharmaceutical–technological insight with pharmacotherapeutic knowledge to cater to specific patient needs. Product knowledge is essential for responding to clinical enquiries. It is also essential for making informed purchasing decisions and for ensuring safe working conditions in a pharmacy environment, particularly in managing health risks for staff handling or preparing medicines [1].

A comprehensive six-year university programme, integrating scientific, practical, and clinical training, equips future pharmacists in the Netherlands with the knowledge and competencies required to meet professional standards [1,17]. The Royal Dutch Pharmacists Association (Dutch: Koninklijke Nederlandse Maatschappij ter bevordering der Pharmacie; KNMP) supports the professional field through standards, guidelines, knowledge bases, and helpdesks. Information and guidance are aligned with the latest scientific and practice-oriented developments [18,19]. Specific professional support for hospital pharmacists is provided by the Dutch Association of Hospital Pharmacists (Dutch: Nederlandse Vereniging voor Ziekenhuisapothekers; NVZA) [20].

This article aims to provide a comprehensive overview of the current state of extemporaneous compounding, pharmacy preparations and related product care within the pharmaceutical profession and healthcare system in the Netherlands. Topics presented and discussed include the types of pharmacy preparations and clinical needs, the organisation around pharmacy preparations in community and hospital pharmacies, the regulatory and legal framework, outsourcing, quality assurance, education, and international benchmarking. Specific topics linked to pharmacy preparations, often with a research component and a strong focus on product care, are highlighted: optimising individual treatment, specialist product groups, and technological innovations. Paediatric dosage forms, swallowing difficulties and feeding tubes, hospital-at-home care, reconstitution of oncolytic drugs and biologicals, total parenteral nutrition (TPN), advanced therapy medicinal products (ATMPs), radiopharmaceuticals and optical tracers, clinical trial medication, robotisation in reconstitution, and patient-centric solid oral dosage forms are addressed. Good practices are highlighted and examples from practice are given.

## 2. Rationale of Pharmacy Preparations

### 2.1. What to Dispense

**Figure 1:**
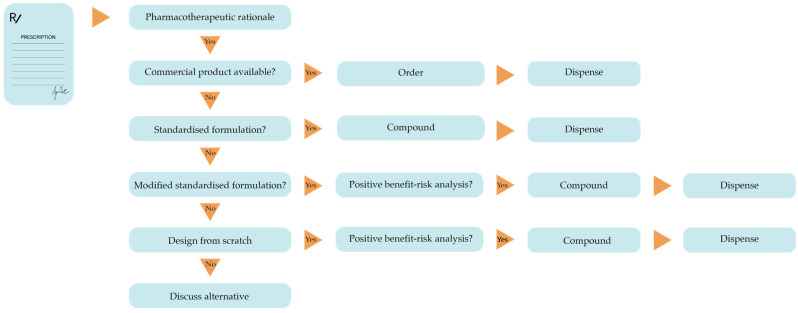
Decision tree for the medicinal product to be dispensed to a patient upon receipt of a prescription from a healthcare provider; based on [21].

Upon receiving a prescription from a healthcare provider, the pharmacist is responsible for verifying the pharmacotherapeutic rationale, a prerequisite for dispensing any medicinal product to a patient. In most cases (>95% of all prescriptions), the first choice is a licensed commercially available product [5]. If such a product is not available, or unable to provide the best solution for the patient, a standardised pharmacy preparation is preferred. If that is also not available, a non-standardised pharmacy preparation may be considered. A non-standardised pharmacy preparation can be an unvalidated modification of a standardised preparation while adhering to general and established procedures for preparing specific dosage forms (see Section 5.2). It can also be a product formulation that is designed from scratch; however, this carries the highest risk of quality loss. Only a minimal set of quality requirements is available, and the composition, stability, and biopharmaceutical performance may not be scientifically validated. Figure 1 shows the decision tree to be followed.

In all cases, a benefit–risk assessment should be conducted on the pharmacotherapeutic benefit against any potential loss of product quality. In this context, it is important to consider that, in most instances, a non-standardised or self-designed product will be made for a single patient. If the pharmacy preparation does not meet the highest possible quality level but can provide the best therapy to the patient, the benefit–risk assessment may favour it. An alternative, especially in community pharmacy, may be to consider together with the prescriber a different commercially available or a different standardised product as a substitute, provided that the desired pharmacotherapeutic outcome will be similar [21]. In hospital pharmacy, however, it more frequently occurs that the only treatment option for a patient is a substantiated and well-considered ad hoc preparation.

### 2.2. Types of Pharmacy Preparations

Three types of pharmacy preparations are distinguished and covered by Dutch guidelines: (1) products entirely prepared from raw materials, (2) the modification of commercially available products, and (3) the reconstitution of purpose-designed products [22,23,24]. Always, product care is essential to obtain a high-quality and safe product.

Modifying a licensed product beyond its Summary of Product Characteristics (SmPC) yields an unlicensed product, shifting the responsibility for quality from the industrial manufacturer to the compounding pharmacist. Since this practice is associated with pharmaceutical–technological and biopharmaceutical challenges, in-depth product knowledge is required. Examples are pulverising tablets and mixing the powder with excipients to make capsules, and the conversion of an oral solid dosage form into an oral liquid dosage form such as a suspension.

In the case of reconstitution, a licensed product is designed to undergo final handling as specified in the SmPC before it is administered to a patient. A reconstituted product remains licensed [25]. Reconstitution of a product is classified as a manipulation rather than compounding in regulatory frameworks [12,13,14,15,16,22]. However, similar handlings are carried out while maintaining comparable product care measures. Reconstitution is common in both community and hospital pharmacies. Examples include reconstitution of an injection fluid, diluting a concentrate into an infusion bag (admixing), and dissolving a freeze-dried antibiotic in water to obtain an oral liquid dosage form. Reconstitution in excess of an SmPC legally yields an unlicensed preparation [25]. Examples are the use of a reconstitution solution not mentioned in the SmPC, or a differently assigned shelf life.

### 2.3. Justification of Pharmacy Preparations

Choosing a pharmacy preparation is justified when no commercial product is available to meet the patient’s specific needs. Optimal pharmacotherapy can then be achieved with a tailored approach, often improving patient compliance and providing greater flexibility. Dose adjustment, dosage form adjustment, or modification of a formulation are common in this context, especially for children and the elderly. A patient with an allergy or hypersensitivity to a certain excipient can be served with an adapted, compounded formulation. Products with a short shelf life can only be made on demand by extemporaneous compounding. Furthermore, for the production of early-stage investigational medicinal products (IMPs), extemporaneous compounding is needed. Medicine shortage, a current major problem in the Netherlands, may be alleviated with pharmacy preparations. Finally, recent developments such as individualised therapies based on a patient’s genetic profile are expected to increase the demand for patient-tailored medicines that are not commercially available [26,27,28,29,30].

## 3. The Dutch Healthcare System and Pharmacy Preparations

### 3.1. Demographic Data

At the start of 2025, the Netherlands had more than 18 million inhabitants, with a nearly equal share of males and females. The country is facing an ageing population. On 1 January 2025, 54% of the population was older than 40 years and 21% older than 65 years. For comparison, this was 45% and 13%, respectively, on 1 January 2000. The median age in 2020 was 41.5 years; in 2000, it was 36.2 years [31,32]. An ageing population places an increased demand on the healthcare system and necessitates a more personalised approach, including tailor-made pharmacy preparations.

### 3.2. Professional Organisations and Support

The KNMP offers extensive support to daily practice of the pharmaceutical profession in the Netherlands [33]. A key service is the KNMP Knowledge Bank (Dutch: Kennisbank), a digital information resource available via paid subscription for pharmacists and pharmacy teams [21]. It includes services aimed at pharmaceutical product care and compounding provided by the Laboratory of Dutch Pharmacists (Dutch: Laboratorium der Nederlandse Apothekers; LNA). The LNA serves as the knowledge centre for pharmaceutical product care within the KNMP, focusing on ensuring the quality and availability of pharmaceutical products for patients. The LNA conducts research on pharmaceutical raw materials, semi-finished and finished products, dosage forms, and packaging materials for both human and veterinary use, as well as for clinical research [33].

LNA’s services include the Formulary of Dutch Pharmacists (Dutch: Formularium der Nederlandse Apothekers; FNA), LNA Messages, LNA Procedures, Oralia VTGM, Parenteralia VTGM, and RiFaS, the Risk Instrument for Pharmaceutical Substances. Further support is offered by the Special Interest Group (SIG) ‘Product Care and Preparation’ (Dutch: Productzorg en Bereiden), consisting of pharmacists who share commitment to pharmaceutical product care.

The FNA is a collection of nationally standardised formulations for medicinal products for community and hospital pharmacies that produce pharmacy preparations (see Section 5.2).

LNA Messages (Dutch: LNA Mededelingen) are irregularly appearing newsletters from the LNA, to ensure pharmacists stay informed about FNA formulations, LNA procedures, and other LNA resources. These newsletters cover topics such as medicine availability, counterfeit drugs, and pharmacy working conditions, along with background information on new and existing pharmacy preparations. As part of quality assurance, the newsletters support pharmacists in ensuring consistency, safety and efficacy in individual and small-scale preparations.

LNA procedures are instructional documents that outline actions, processes and methods related to pharmaceutical product care. Oralia VTGM provides guidance on the preparation of oral medicines for patients with swallowing difficulties or those requiring administration with a feeding tube. Parenteralia VTGM offers information on the preparation, stability, admixture compatibility, and storage of parenteral medicines (see Section 5.4). RiFaS delivers tailored advice on the safe handling of pharmaceutical substances, taking the available equipment in the pharmacy and the duration of exposure into account [34].

The KNMP has established the Directive Pharmacy Preparations (Dutch: Richtlijn Bereiden) to provide community pharmacists with GMP-based guidelines for high-quality small-scale preparations [35]. This directive outlines protocols covering key aspects including quality control, risk assessment, documentation, and correct labelling of pharmacy preparations.

For hospital pharmacies, support is also provided by the NVZA, in addition to the KNMP [19]. A key NVZA document is the GMP-Z (Good Manufacturing Practice for hospital pharmacies) [21], which serves as the foundational standard for the preparation of medicinal products in hospitals. The NVZA has dedicated working groups focused on pharmaceutical product care within the hospital settings and is internationally affiliated with the European Association of Hospital Pharmacists (EAHP) [36]. GMP-Z is aligned with the requirements of the Health and Youth Care Inspectorate (Dutch: Inspectie Gezondheidszorg en Jeugd; IGJ) [22,37].

### 3.3. Community Pharmacy

Primary care in the Netherlands, which is primarily focused on outpatient services, included 1953 community pharmacies as of 1 January 2024 [38]. In 2023, 2.8% of all medications dispensed by these pharmacies were compounded preparations, with topical and oral products accounting for the majority, at 53% and 33%, respectively [38,39]. Over the past decades, compounding in community pharmacies has declined significantly. Today, only around 125 pharmacies regularly perform in-house extemporaneous compounding activities [40], while the remainder fully outsource these tasks to specialised compounding pharmacies. This shift is largely driven by the high costs, as well as the need for specialised resources, trained personnel, and infrastructure required to meet strict regulatory and quality standards. However, despite the decline in extemporaneous compounding, reconstitution remains a routine practice in all community pharmacies.

A compounding community pharmacy should have a dedicated compounding area that meets defined standards for cleanliness, organisation, and safety. This includes space for the appropriate storage of raw materials and compounded medications, where applicable in validated refrigerators. A separate, designated area must be available for the cleaning and sanitisation of equipment to prevent cross-contamination, especially when preparing multiple formulations or handling hazardous substances. Basic compounding equipment includes balances, mortars and pestles, mixing spatulas, a capsule filling and closing apparatus, a dust extraction cabinet, hot plates or other heating devices, homogenisers and mixers, a laminar flow cabinet, and an autoclave. Pharmacy technicians involved in compounding must possess specialised qualifications (see also Section 3.5).

Compounding pharmacy software is used to manage and streamline various operational processed, including inventory control, formulation management, and the weighing of active pharmaceutical ingredients (APIs) and excipients. It improves efficiency, supports compliance with regulatory standards, ensures accuracy, and ultimately enhances patient safety.

Table 1 presents the most common types of pharmacy preparations produced in compounding community pharmacies in the Netherlands.

After compounding and dispensing a pharmacy preparation, the pharmacy submits a reimbursement claim to the patient’s health insurance company. All Dutch citizens are required to have health insurance. The reimbursement procedure typically involves three steps. First, the medical necessity is verified based on the prescription and the patient’s medical condition. Second, a cost assessment is conducted, which includes evaluating the price of raw materials, labour costs for compounding, and any additional pharmacy overhead. Insurance companies may negotiate reimbursement rates or set maximum limits for compounded products. Third, a claim is either approved or denied. If the compounded medication does not meet the insurer’s criteria, the reimbursement claim is denied, and the patient must cover the full cost. Decisions on reimbursement claims can be postponed for up to two years after dispensing, which exposes compounding pharmacies to significant financial risks [41].

### 3.4. Hospital Pharmacy

Hospital pharmacists primarily manage specialised pharmaceutical patient care for patients in general hospitals (secondary care) and specialised, often academic, hospitals (tertiary care). In 2025, the Netherlands had 69 hospital organisations, including 8 academic medical centres, 113 hospital locations (academic, general and children’s hospitals), and 137 outpatient clinics. While the number of hospital organisations is decreasing due to mergers, the total number of locations providing hospital care has remained relatively stable owing to a growing number of outpatient clinics [42].

In all Dutch hospital pharmacies, the reconstitution of parenteral products is routine daily practice. Many, but not all hospitals also prepare medications for individual patients. These preparations can, in principle, encompass the full range of dosage forms. However, stock preparations are prepared in only a limited number of hospital pharmacies, primarily focusing on sterile and aseptic products (syringes, infusions, ampoules) as well as a selection of non-sterile products (oral liquids, oral drops, cutaneous preparations) that are not commercially available. In cases where pulmonary drug administration is requested, the compounding of pressurised metered dose inhalers or dry powder inhalers is not feasible, because of technical requirements related to their production and quality control. However, aqueous solutions suitable for nebulisation can be prepared by hospital pharmacies or specialised compounding pharmacies. When compounding such solutions, it is essential that quality attributes such as the particle size of the aerosol and output rate are determined, requiring specialised equipment which is not generally available in most pharmacies [43].

The NVZA issues the professional guideline GMP-Z, which hospital pharmacies are required to follow. Introduced in 1996, GMP-Z is regularly revised and updated to reflect advancements in hospital pharmacy practice [20,22].

Like community pharmacies, hospital pharmacies also outsource pharmacy preparations, either to centralised compounding facilities or to other hospital pharmacies. These facilities must fully comply with GMP manufacturing requirements and are required to obtain a ‘toleration declaration’ (Dutch: gedoogverklaring) from the IGJ [44]. Some of these facilities hold a GMP manufacturing license (Manufacturing and Importation Authorisation (MIA); Dutch: fabrikantenvergunning) for producing pharmacy preparations for third parties [45].

An alternative to outsourcing patient-specific preparations is the ‘carry-over’ regulation, whereby the prescription is transferred from the dispensing to the compounding pharmacy. The compounding pharmacy takes responsibility for preparing the medication, while tasks such as dosage verification and interaction checks may remain with the dispensing pharmacy, depending on the terms of the contract. This regulation, which does not require a toleration declaration, is particularly useful for cytostatic drugs, radiopharmaceuticals, and medications for metabolic diseases [46].

In hospital pharmacies, both parenteral individual and stock preparations are produced in cleanrooms that comply with GMP [12,47] and GMP-Z [22] requirements. Due to the wide range of products handled, different items are often manufactured on the same production line and/or within the same working area. This imposes stringent demands on the cleanroom type, cleaning and disinfection procedures, cross-contamination prevention strategies, and personnel qualifications. Particular emphasis is placed on personnel training, especially in aseptic procedures and quality assurance. Staff are required to constantly develop and maintain their skills through periodic requalification to demonstrate ongoing competence [22]. In larger hospital pharmacies, both sterile and non-sterile compounding from raw materials to produce individual and intermediate-scale stock preparations is common practice.

Hospital pharmacies play a crucial role in the reconstitution of complex medications, particularly sterile products such as chemotherapy, immunotherapy, TPN, ready-to-use (RTU) and ready-to-administer (RTA) syringes. They are also involved in preparing personalised medicines for specific patient groups, such as paediatric patients and those with swallowing difficulties (see also Section 7.1.1 and Section 7.1.2).

To further enhance patient safety, there is a growing demand for RTA and RTU products prepared by hospital pharmacies. This approach has been shown to reduce medication errors and prevent microbial contamination [48,49]. One way to ensure the availability of RTA products on hospital wards is to have pharmacy assistants prepare them in dedicated rooms located on or near the ward. Alternatively, preparation can be centralised in the hospital pharmacy, where the syringes are aseptically filled. However, these syringes have a limited shelf life of weeks and must be stored refrigerated, which poses challenges for storage capacity in larger-scale production. Consequently, hospital pharmacies have increasingly developed and implemented the production of prefilled sterilised syringes (PFSSs) in recent years. PFSSs offer the advantage of a long shelf life (up to three years) at room temperature, along with high quality and an efficient, automated production process that saves time. Approximately one-third of parenterally administered medications in a hospital are suitable for delivery in RTA formulations. This can significantly improve medication safety and saves time for nursing staff [48,49,50].

Inpatient medication costs, including those for pharmacy preparations, are covered by the hospital’s central budget. As a result, hospital pharmacies calculate internal cost prices and develop business cases for reconstitution activities, with quality improvement being a key factor in the decision to undertake these services.

### 3.5. Pharmacy Technicians and Pharmacy Assistants

Pharmacy technicians and assistants play a vital role in Dutch pharmacies. To become a certified pharmacy technician, individuals must complete a three-year vocational programme covering topics such as diseases, medication effects, patient communication, pharmaceutical calculations, and quality assurance [51]. Graduates earn the title of pharmacy technician and are eligible for registration in the public register for healthcare professionals (see also Section 4.1) [52,53]. In addition to technicians, most pharmacies also employ pharmacy assistants who do not undergo the same formal education. Instead, they are trained either on the job or through shorter, less comprehensive training programmes, including final examinations, as offered by several commercial course providers [54,55]. The average of pharmacist-to-technician/assistant ratio in Dutch community pharmacies is estimated at approximately 1:7, though this varies between pharmacies [40].

Pharmacy technicians handle prescription verification, medication dispensing, patient counselling, and the preparation and reconstitution of medicines. Pharmacy assistants perform logistic tasks, support pharmacy technicians, and help at the pharmacy’s production site.

Pharmacy technician training has increasingly focused on communication, clinical pharmacy services, and patient counselling, leaving graduates with limited preparation and reconstitution skills. As a result, employers must provide additional training, including a supervised onboarding programme and commercial e-learning modules [55,56].

Training follows a three-stage process: observation, hands-on practice under direct supervision, and independent execution under indirect supervision. Upon completion, a pharmacy assistant is qualified to perform one or more specific tasks. For aseptic handling, a KNMP guideline [57] outlines the initial person-specific qualification, which involves completing 30 individual handling steps using a culture media fill protocol. This qualification must be renewed at least annually. While not mandatory, it is considered good practice to implement an additional requalification protocol for both pharmacy technicians and assistants. This may include the periodic production of worst-case preparations and the requirement for a minimum number of hours or days per month or year which staff should perform preparation tasks [58].

Excluding preparation and production-related content from pharmacy technician training limits their fundamental understanding of pharmaceutical compounding. While on-the-job training can teach individuals to perform tasks correctly and follow established protocols, a lack of insight into the underlying scientific principles and rationale increases the risk of errors. Furthermore, reliance on an educational model that emphasises simply ‘repeating what others already do’ may restrict the capacity for innovation and hinder the ability to adapt to new developments. In the long term, this could pose a threat to the sustainability of the current system.

In addition to pharmacy technicians, there is a growing trend towards employing pharmaceutical managers (Dutch: farmakundigen) in pharmacies. These professionals act as intermediaries between pharmacy technicians and (hospital) pharmacists. Their training enables them to support pharmacists in specialised tasks such as organising production workflows, planning work schedules, and assisting with product development and financial management. They may also become involved in overseeing compounding-related processes and their quality assurance [59].

The pharmacist retains ultimate responsibility for the quality of the preparations produced in the pharmacy. The pharmacist is also responsible for providing adequate training programmes to ensure that pharmacy staff are properly qualified, as well as for ensuring the availability of appropriate infrastructure, compounding equipment, standard operating procedures, and production protocols.

## 4. The Dutch Regulatory and Legal Framework for Pharmacy Preparations

### 4.1. The Position and Qualification of the Dutch Pharmacist

Two specific activities are exclusively reserved for pharmacists registered in the Netherlands: dispensing and preparing medicines [60]. The legal and regulatory framework surrounding pharmacy preparations is defined by various national laws and guidelines, which are currently being aligned with updated EU pharmaceutical legislation [61]. According to the Medicines Act (Dutch: Geneesmiddelenwet; GW) [62], which governs the manufacture, distribution and sale of medicines, pharmacists are legally permitted to prepare medication for patients in their own pharmacy upon receiving a health practitioner’s prescription. A prerequisite is that the pharmacist must be competent to perform this task through appropriate education, training, and practical experience. In addition, all preparation activities must adhere to strict standards of safety, quality, and hygiene. The Medical Treatment Contracts Act (Dutch: Wet op de Geneeskundige Behandelingsovereenkomst; WGBO) [63] establishes that pharmacists are co-responsible for a patient’s treatment, together with physicians. This shared responsibility may result in deciding that the preparation of an extemporaneous, non-commercially available medication is the most appropriate therapeutic option for a specific patient.

Pharmacists who are actively working in healthcare in the Netherlands must be registered in a public register for healthcare professionals (the so-called BIG register), according to the Professions in Individual Health Care Act (Dutch: Wet op de Beroepen in de Individuele Gezondheidszorg; Wet BIG) [53,60].

### 4.2. Extemporaneous Pharmacy Preparations

One of the primary objectives of the general EU pharmaceutical legislation is to ensure the safety, efficacy and quality of medicines available on the EU market [64,65]. As such, the manufacturing, distributing and marketing of medicines require appropriate licenses. However, extemporaneous pharmacy preparations are exempt from these licensing requirements, as they are considered unlicensed products.

In the Netherlands, two situations regarding pharmacy preparations are distinguished. The first involves medicines prepared within a pharmacy for direct supply to patients of that same pharmacy (see Section 4.2.1). The second concerns the supply of compounded medicines from one pharmacy to other, commonly referred to as pharmacy-to-pharmacy delivery or outsourcing (see Section 4.2.2).

#### 4.2.1. Medicines Prepared in a Pharmacy for Own Patients

The general rule in the Netherlands is that manufacturing and supplying medicines without the appropriate manufacturing licenses is prohibited. However, this requirement does not apply to medicines compounded by a pharmacist (or by a qualified pharmacy assistant or technician under the responsibility of a pharmacist) within a pharmacy, provided the medicine is intended for direct supply to a patient of that specific pharmacy. This exception is derived from Article 3 of Directive 2001/83/EC [64].

In a letter to the Dutch Parliament on 8 April 2019 [66], the Minister of Healthcare outlined the conditions under which a pharmacy preparation is allowed. Based on the Medicines Act [62], these conditions are as follows:

- The preparation takes place in the pharmacy, based on a medical prescription for an individual patient or as stock for patients yet to be determined by that pharmacy;
- The preparation complies with the Ph. Eur.;
- The preparation is intended for dispensing on a small scale.

The third condition, dispensing on a small scale, is subject to a numerical criterion, intended to provide clarity to stakeholders. As defined by the Minister of Healthcare, small-scale dispensing means:

- Dispensing to up to approximately 50 unique patients per month for long-term use of the medication;
- Dispensing to approximately 150 patients per month for short-term use.

Short-term use is one week or less, while long-term use is more than one week.

If all of the above-mentioned conditions are met, pharmacists are permitted to compound on a small scale for their own patients, even when a medicinal product with marketing authorisation is available on the market. However, it should be noted that the numerical criteria have not yet been included in a generally binding regulation.

The European GMP guidelines [12] are primarily designed for the industrial manufacture of medicines. However, their principles are also relevant to all pharmaceutical preparation processes carried out in community and hospital pharmacies. Since these guidelines do not provide sufficient detail for the preparation of medicines in pharmacies, the KNMP and the NVZA have developed supplementary GMP-based guidelines specially tailored to pharmacy settings to ensure the quality of pharmacy preparations. These include the following:

- KNMP: Directive Pharmacy Preparations, for community pharmacies [35];
- NVZA: GMP-Z, for hospital pharmacies [22].

The IGJ is the supervisory authority responsible for enforcing of these guidelines, thereby safeguarding the quality and safety of medicines prepared in pharmacies throughout the Netherlands [37].

#### 4.2.2. Pharmacy-to-Pharmacy Delivery and Outsourcing

As outlined in Section 4.2.1, pharmacies in the Netherlands are generally only permitted to compound for their own patients, and in small quantities. However, societal and professional developments have made it increasingly difficult to maintain operational compounding facilities, expertise, and proficiency in every community and hospital pharmacy. In addition, focus clinics and other care institutions without an in-house pharmacy also need compounded products. At the beginning of the 21st century, this insight led to a consensus among the government, the IGJ and pharmacists that a different model was necessary. The goal was twofold: to ensure continued patient access to necessary compounded medicines, and to enable compounding at a larger scale, thereby justifying the considerable investments in facilities, quality systems, training and expertise.

The supply of compounded preparations from one pharmacy to another and the outsourcing compounding to specialised pharmacies, also known as ‘collegial supply’, has been fully regulated since 2017 [67]. This was outlined in the IGJ document ‘Circulaire handhavend optreden bij collegiaal doorleveren van eigen bereidingen door apothekers’ (translation: Circular on enforcement action in the event of collegial forwarding of own preparations by pharmacists; hereafter referred to as the ‘IGJ circular’). The IGJ formulated six restrictive conditions under which pharmacy-to-pharmacy delivery is permitted [68] (see Table 2). If all conditions are met, no enforcement action is taken (so-called ‘tolerance policy’).

The quantitative restrictions for small-scale compounding by pharmacies for their own patients do not apply to pharmacy-to-pharmacy delivery and outsourcing arrangements.

As of 1 February 2025, the IGJ circular [68] was replaced by a policy rule with a legal base in Dutch administrative law [71,72,73]. The policy rule maintains the approach that pharmacy-to-pharmacy delivery of compounded products is permitted without marketing authorisation, provided that the specified conditions are met (Table 2). The policy rule may contribute to the ongoing revision of the EU directive and regulation on medicinal products for human use [74].

In 2025, the outsourcing landscape in the Netherlands has evolved to include approximately 20 specialised compounding pharmacies operating under various legal structures. Unlike in some other countries, there are no legal restrictions on ownership. Both pharmacies and private companies are active in supplying compounded products to community pharmacies, hospital pharmacies, focus clinics, and outpatient services.

These pharmacies generally follow established FNA formulations and procedures or adopt a quality-driven approach in the development of new formulations. When supported by appropriate quality measures, outsourcing ensures that compounded products are prepared in accordance with GMP standards and meet the required quality criteria.

The product portfolios of central compounding pharmacies primarily consist of non-sterile products, although there is a growing trend towards ophthalmic preparations and patient-specific sterile products for homecare. Additionally, the available expertise and infrastructure of these facilities are often leveraged to manufacture orphan drugs.

Hospital-based compounding facilities primarily serve hospital pharmacies. Their portfolios typically include parenteral products, supplied in RTA or RTU formats, which help reduce medication errors and ease the burden on nursing staff by eliminating the need for bedside preparation of infusions and injectables. Furthermore, the available expertise and facilities are often used to manufacture IMPs for investigator-initiated clinical trials (see Section 7.2.5).

Both public institutions and private companies use their infrastructure to mitigate drug shortages. Examples are ipratropium bromide inhalation fluid, pethidine hydrochloride ampoules, and suxamethonium chloride ampoules. Recently, in response to global supply chain disruptions following the destruction of a major production site in the USA caused by hurricane Helene in October 2024, Dutch hospital pharmacies produced sodium chloride and glucose infusion bags to address the resulting shortages [75,76].

### 4.3. Enforcement

The IGJ monitors compliance with pharmaceutical standards and regulations [37,62,68,72]. Pharmacy inspections or audits are conducted to assess adherence to the required standards related to cleanliness, equipment, procedures, and personnel qualifications involved in pharmacy preparations. These inspections also ensure compliance with the European GMP standards or applicable derived guidelines. The IGJ recognises the GMP-Z guidelines as the standard for pharmaceutical compounding in Dutch hospital pharmacies. The GMP-Z was developed and is maintained in collaboration with the IGJ, and any update to the guideline only takes effect following IGJ approval [22,37].

### 4.4. International Context

In most European countries, extemporaneous compounding is an integral part of routine pharmacy practice, particularly when commercial products are unavailable or inadequate to meet specific patient needs [7,10,24]. Compounding practices in Dutch pharmacies share many features with general European approaches while also reflecting distinctive national characteristics. Europe does not have a unified regulatory framework for pharmacy compounding, but the legal framework governing medicinal products excludes pharmacy preparations from its scope, meaning that marketing authorisation and manufacturing authorisation are not required. Additionally, in cases of specific medical needs, individual EU member states are allowed to establish their own regulations regarding the use of unlicensed products [64].

In general, EU countries recognise that extemporaneous compounding is a competence reserved to pharmacies that dispense medicines directly to patients. The percentage of pharmacies that actually perform in-house compounding activities varies across European countries and is influenced by whether outsourcing is permitted [77,78,79]. However, reliable benchmarking data on this matter are not readily available.

There is always great emphasis on the quality and safety of compounded medication, but there are differences in the extent of standardisation of formulations (see Section 5.2). The Netherlands and Germany (Deutscher Arzneimittel-Codex/Neues Rezeptur-Formularium (DAC/NRF; translated: German Drug Codex/New German Formulary) [80]) have a well-developed regulatory framework in this respect, while in other European countries and in the USA, compounding practices are less streamlined.

The EAHP plays a key role in promoting and guiding pharmaceutical compounding practices within European hospitals by setting standards, developing policy and guidelines, providing training programmes, and supporting research and innovation [7,36].

In most European countries, outsourcing is typically not permitted. However, in some northern European countries, including the Netherlands, Belgium, Germany, Sweden, Ireland, and the United Kingdom, pharmacies are allowed to outsource the preparation of compounded or unlicensed products within defined national legal frameworks. These frameworks usually stipulate that outsourcing is only permitted when no licensed product can adequately meet a patient’s therapeutic needs [7,10,24]. Outsourcing facilities generally operate on a larger scale, and GMP requirements for such operations vary by country.

The USA employs a more commercialised system compared to Europe, with larger outsourcing facilities, alongside traditional compounding pharmacies [81]. The U.S. Food and Drug Administration (FDA) regulates pharmaceutical compounding to ensure patient safety and product quality [8]. Compounding pharmacies are categorised into two distinct types under Sections 503A and 503B of the Federal Food, Drug, and Cosmetic Act. [81].

Pharmacies operating under Section 503A focus on preparing customised medications for individual patients based on specific prescriptions. These are typically traditional compounding pharmacies, often located in community or hospital settings. Section 503B, introduced in 2013 in response to safety concerns, created the category of ‘outsourcing facilities’, which are permitted to produce compounded medications at a larger scale and must comply with stricter GMP standards [81]. This dual regulatory structure enables the U.S. healthcare system to meet a broad range of patient and clinical needs while ensuring the quality and safety of compounded medicines [82].

## 5. Quality Assurance of Pharmacy Preparations

### 5.1. Quality by Design (QbD)

Licensed and unlicensed medicinal products are prepared under different conditions, which has implications for their quality and quality assurance. Industrially manufactured products are required to meet the highest possible quality standards along with evidence of product safety and efficacy, as this is required for registration.

Pharmaceutical companies commonly adopt the QbD approach in product development to ensure the quality of medicines. QbD is a systematic, proactive methodology that focuses on understanding and controlling variability in materials, processes, and formulations to maintain consistent product quality, safety, and efficacy. By managing all variables from the outset, QbD ensures that each batch consistently meets the required quality standards. Regulatory agencies such as the EMA and the FDA strongly support the use of QbD, making it a standard practice in the pharmaceutical industry [83,84].

While the QbD approach enhances product quality, it can also lead to longer development timelines and higher production costs. The scale of production generally determines the extent of substantiation required for the pharmacotherapeutic and technical design. In the context of small-scale preparations, QbD is generally not feasible. Nevertheless, although small-scale production is more customised and less standardised than large-scale manufacturing, pharmacotherapeutic and technical design can still be applied using standardised formulations.

### 5.2. Standardisation of Formulations

The KNMP supports the professional field by providing standardised formulations that are not commercially available but needed in daily practice. The LNA, a division of the KNMP, developed and maintains the FNA, a comprehensive formulary containing standardised formulations and guidelines for compounded preparations, accessible through the KNMP Knowledge Bank [21,33].

On 1 May 2025, the FNA included 226 nationally standardised formulations for pharmacy preparations [85], which can be produced in community, hospital, and specialised compounding pharmacies. Each formulation includes detailed information on the qualitative and quantitative composition, preparation method, recommended packaging, storage conditions, shelf life, and quality requirements. Extensive background information, including reference to scientific literature, underpins the formulation design and preparation method, along with a pharmacotherapeutic assessment of each preparation. Shelf life and usage periods are generally based on real-life stability testing. For most FNA preparations, analytical protocols have been established, including methods for qualitative and quantitative analysis of active substances and excipients. Where relevant and feasible, information on the degradation of active substances is included. The LNA develops its analytical protocols based on pharmacopeial standards or through in-house development of non-standardised methods [21,33].

Because both the design of the composition and the preparation methods are carefully developed and validated, the formulations are robust. When prepared by a trained professional (pharmacist or pharmacy assistant), the quality of the final product will be assured and consistent, resulting in reproducible outcomes. Consequently, the responsible pharmacist can guarantee the quality of such compounded preparations.

Regular updates and ongoing maintenance of the FNA are essential to ensure that pharmacists have access to the most current and reliable information on individual and small-scale formulations. New formulations are added to the FNA for various reasons, for instance, when licensed medications are unavailable in the appropriate dose for paediatric patients (e.g., Fluoxetine capsules 5 mg, Hydrochlorothiazide 0.5 mg/mL oral solution). The FNA also provides formulations for medicinal products that are no longer commercially available (e.g., Metoclopramide hydrochloride suppositories 10 mg) or to improve and standardise commonly used preparations (e.g., Clobetasol propionate mouthwash 0.25 mg/mL) [21].

For preparations not described in the FNA, the LNA has developed standardised procedures that provide comprehensive guidelines and validated methods for various dosage forms. The procedures include specifications for formulation design, preparation methods and equipment, raw material selection, and quality control measures.

### 5.3. Product File and Production Batch Record

For each product prepared in a (compounding) pharmacy or hospital pharmacy, a product file is compiled in advance to support and substantiate the pharmacy preparation. The product file contains the pharmacotherapeutic rationale and all information pertaining to the preparation and the manufacturing process. It covers specifications for the API and excipients, batch size, composition, preparation method, materials and equipment used, in-process controls and final quality controls, test results of trial preparations, storage conditions, shelf life, packaging, labelling, and patient information [86].

For each product to be made, a production batch record (protocol) should be available. This is derived from a master file, which is supported by the corresponding product file. A master file serves as a template for the production batch record for each individual batch or product. The release of the preparation by an authorised pharmacist is based on a completed production batch record containing all the data recorded during and after preparation. This document is archived for traceability and accountability. The entire procedure follows the principles of GMP.

### 5.4. Preparation for Administration and Reconstitution

In addition to supporting the preparation of compounded medicines, the LNA provides guidance on the preparation of licensed medicines for administration. Two dedicated databases are accessible via the KNMP Knowledge Bank: Oralia VTGM and Parenteralia VTGM [21]. VTGM is a practice meaning ‘voor toediening gereedmaken’ in Dutch, which can be translated as ‘preparation handlings needed for administration’. A prepared VTGM product is considered an RTA product, requiring no further handling prior to administration to a patient.

Oralia VTGM contains more than 600 individual monographs that offer practical guidance for the safe administration of medicines to patients with swallowing difficulties and those who require a feeding tube. Designed for pharmacists and pharmacy staff, the database consolidates all available information into a single resource. Each monograph is primarily based on product information, data provided by pharmaceutical companies, the scientific literature, and practical research. The monographs provide guidance on the safe handling of specific medicines and formulations. Where possible, a licensed formulation is recommended, but the monographs also suggest alternative administration routes and substitute medicines to consider. If no alternatives are available, the monographs provide recommendations for safe handling of the medicinal product, including instructional videos. Table 3 illustrates the typical content of a monograph in Oralia VTGM.

In addition to the Oralia VTGM database, another dedicated website, Oralia.nl [87], has been developed for healthcare professionals other than pharmacy staff, such as nurses. It presents the same essential information as Oralia VTGM, but in a simplified format, omitting technical details that are not relevant to nursing practice, such as the explanation of why a medicine can or cannot be processed.

Parenteralia VTGM provides fundamental information on processing licensed medicines intended for parenteral administration. It includes data on stability, raw materials, and compatibility, as well as specific product-related details such as preparation instructions and the final concentrations. Serving as a reference source, it enables pharmacists to answer daily queries regarding parenteral medicines. Nurses require direct instructions on how to prepare, administer, and store specific medicines but have no direct access to the information on the KNMP Knowledge Bank [21]. Therefore, (hospital) pharmacists can use Parenteralia VTGM to develop monographs and instructions tailored to the needs of nursing staff.

Parenteralia VTGM covers all commercially available parenteral medicines, as well as those for which an FNA formulation exists. However, due to their specific nature, no VTGM monographs are available for cytostatic drugs, (X-ray) contrast agents, radiopharmaceuticals, dialysis fluids, and additives for TPN. Additionally, the database includes VTGM products for 50 medicines intended for paediatric patients from one month of age. For consistency, the format is aligned as closely as possible with that of adult VTGM monographs. However, paediatric preparations often require dilution due to the higher concentrations present in the available commercial products. The database also provides calculation examples for pump setting and/or starting doses. The information in Parenteralia VTGM is organised by active substance and presented in the form of monographs (see Table 4).

### 5.5. Raw Materials

Pharmacy preparations should only be manufactured with pharmaceutical-grade raw materials, with sufficient knowledge of their origin and quality, meeting pre-set requirements.

Active substances and excipients used in pharmacy preparations must be manufactured in accordance with EU GMP part II and must comply with the Ph. Eur. monograph ‘Substances for pharmaceutical use’ [6,12]. Where specific monographs exist for individual raw materials, their quality must also meet the requirements outlined in those monographs. If the Ph. Eur. provides no or insufficient information, the British Pharmacopoeia (BP), the United States Pharmacopeia (USP), the Japanese Pharmacopoeia (JP) or another reputable pharmacopoeia should be consulted. In the absence of any applicable monograph, the required quality of the raw materials must be defined and documented based on a risk assessment tailored to their intended use and associated risks [88]. If insufficient knowledge is available, the pharmacist should refrain from using such raw materials for production.

For licensed medicinal products, the quality requirements of the active substance are evaluated by a regulatory authority such as the EMA, as part of the Marketing Authorisation Application (MAA) procedure. The active substance manufacturer provides all pivotal information to the Marketing Authorisation Holder (MAH), while the full dossier, including confidential details, is submitted directly to the regulatory authority. This process protects the manufacturer’s intellectual property while ensuring that the MAH retains full responsibility for the quality of the active substance [89].

In contrast, for pharmacy preparations, the licensed pharmacist is responsible for assessing the quality and suitability of the raw materials used, supported by a documented risk assessment. While most information can be obtained from the active substance manufacturer, confidential details typically reviewed by regulatory authorities are often unavailable to the pharmacist.

It is strongly recommended that active substances used in pharmacy preparations be accompanied by a Certificate of Suitability to the Monographs of the European Pharmacopoeia (CEP) or a certificate of analysis referring to the Ph. Eur. [90]. A CEP certifies that the quality of a substance can be adequately controlled using a relevant Ph. Eur. monograph. As part of the CEP procedure, all information about the active substance is submitted independently from an MAH to the European Directorate for the Quality of Medicines and HealthCare (EDQM). The EDQM evaluates whether the substance complies with the applicable Ph. Eur. monograph, along with any additional applicable requirements. Once approved, a CEP is issued for the substance. If a raw material cannot be procured with a CEP, it is recommended that the compounding pharmacist performs a due diligence investigation on the quality and purity of the substance and on GMP standards at the manufacturing site.

Using active substances with a CEP allows pharmacists to rely on the certificate as a proof of quality, thereby reducing the need to evaluate detailed manufacturing and quality control data themselves. While CEP-certified substances help ensure quality, pharmacies must still maintain a comprehensive pharmaceutical quality system (PQS) to effectively manage their raw material suppliers. It is important to note that CEP certificates are not available for all substances listed in the Ph. Eur. As a result, the suitability of the raw material often still needs be assessed by the pharmacist.

In contrast with industrial-scale pharmaceutical companies, hospitals and compounding pharmacies may lack the resources to implement extensive supplier management and qualification systems. Nevertheless, a certain level of vendor management is also required for small-scale pharmaceutical production. In the Netherlands, the KNMP, in close collaboration with the NVZA, has established a vendor management programme aimed at conducting audits at all major supplier sites for APIs, excipients, and packaging materials. The outcomes of these audits are available to members of both organisations and can serve as a valuable tool for evaluating vendors [91].

It is also important to note that many hospital pharmacies are included in vendor management processes conducted by the pharmaceutical industry itself. Participation in clinical trials often results in quality assurance (QA) involvement from the pharmaceutical industry, particularly concerning GMP and Good Clinical Practice (GCP) regulations. The capacity to carry out these tasks must be established and should be included in the costs charged to the sponsor of the clinical trial. Ideally, revenue generated from GMP-related tasks in sponsored clinical trials should be reinvested into the organisation to support its own QA and GMP infrastructure and personnel [22].

Sourcing raw materials can also be challenging, particularly for low-volume orders. Specialised companies can assist with sourcing, qualification, and analysis of raw materials. They provide high-quality raw materials, along with the necessary qualification documentation, supply chain transparency, and supplier qualifications.

## 6. Pharmacy Education in the Netherlands

### 6.1. Universities and Pharmacy (-Related) Programmes

After secondary school, students who pass their state exams in mathematics, physics and chemistry are eligible to study pharmacy or pharmaceutical sciences. Four universities in the Netherlands offer such degree programmes (Table 5): Leiden University (LU), the University of Groningen (UG), Utrecht University (UU) and the Vrije Universiteit Amsterdam (VU). All Bachelor of Science (BSc) programmes are three-year programmes (180 ECTS (European Credit Transfer System)), whereas the connecting Master of Science (MSc) programmes are either two years (120 ECTS) for pharmaceutical sciences, or three years (180 ECTS) for pharmacy.

Approximately 200 to 250 students qualify as pharmacists each year [92], for which they must obtain the MSc Pharmacy degree. The BSc Pharmacy programmes give automatic access to this Master’s programme; BSc programmes in pharmaceutical sciences either only provide indirect access (usually with additional requirements) or no access at all. Ordinarily, students who have completed such a BSc programme will instead continue in one of the MSc programmes in pharmaceutical sciences, which are generally more research-oriented.

### 6.2. Alignment with the Professional Field

The content of the Dutch MSc Pharmacy programmes is initially dictated by the Besluit opleidingseisen apotheker (Decree on training requirements for pharmacists) [93]. This educational decree details the core knowledge and skills a newly qualified pharmacist should have, as well as the subjects the programme should cover and additional stipulations, such as mandatory pharmacy internships.

In 2016, the LU, UG and UU, together with the KNMP, composed the Pharmacist Competency Framework & Domain-specific Frame of Reference for the Netherlands [1]. These documents not only expand on the basic educational decree, but also take the wishes and demands of the professional field into account, thereby closely aligning the education of Pharmacy students with professional practice.

**Figure 2:**
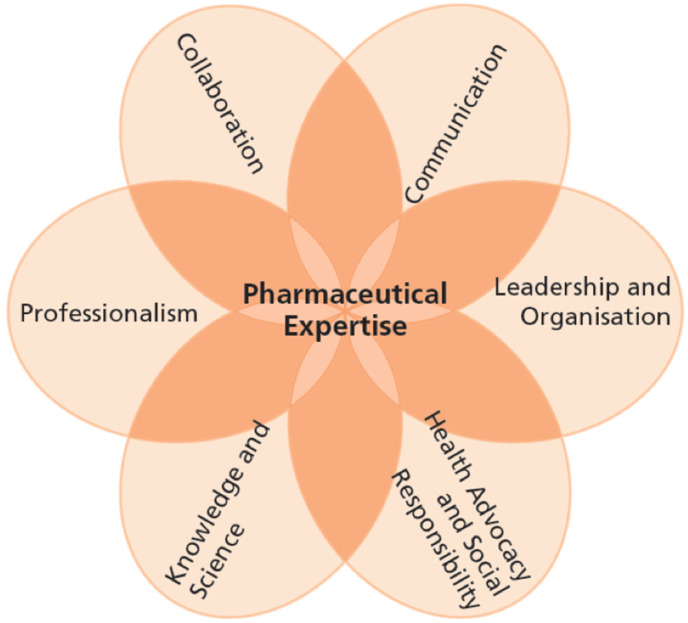
CanMEDS model for the competencies of a Dutch pharmacist. Taken from [1] with permission.

The Frame of Reference outlines the pharmacist’s areas of responsibility and identifies important developments in the field of pharmacy, whereas the Competency Framework defines the learning outcomes for the Bachelor’s and Master’s Pharmacy programme within the context of the Frame of Reference. These learning outcomes are described in terms of competencies based on the CanMEDS model [94]. This competency profile consists of seven complementary areas of competence, with pharmaceutical expertise at its core. The remaining six areas are communication, collaboration, leadership and organisation, health advocacy and social responsibility, knowledge and science, and professionalism (see Figure 2). Within the confines of the Competency Framework, each university can design their Pharmacy education, resulting in programmes with distinctive profiles, yet all providing the necessary training required for a newly qualified pharmacist [17].

The Competency Framework is currently under revision, and a new version of the Frame of Reference is expected to be published later in 2025. The revised documents will be expanded to explicitly include pharmaceutical sciences and the corresponding university programmes in order to cover the full scope of the pharmaceutical field more thoroughly. The new developments identified in the Frame of Reference are now described in ‘knowledge domains’ for the students: drug development, manufacturing and product expertise; education; pharmaceutical care on a population level; and pharmaceutical care on an individual (patient) level. The programmes in pharmaceutical sciences will mainly focus on the first two knowledge domains, while the MSc Pharmacy programmes will include all four domains.

### 6.3. Bachelor’s Pharmacy Programme

Pharmacy and pharmaceutical sciences as academic disciplines are positioned in the centre of life sciences. It connects the natural sciences, mainly chemistry, biology and physics, to medical sciences. The current Competency Framework reflects this multidisciplinary nature in over 40 learning outcomes for the Pharmacy Bachelor’s programmes, categorised into three core domains: knowledge and understanding, skills and professional behaviour. These learning outcomes lay the foundation for the development of the competencies defined for the Pharmacy Master’s programmes and should be considered entry requirements.

The Pharmacy programmes cover pharmaceutical preparations and compounding in the broader context of pharmaceutical product care, motivated by the notion that students should become experts on pharmaceutical products to the fullest extent. In the current Competency Framework, six learning outcomes of the Bachelor’s programme deal implicitly or explicitly with pharmaceutical product care (Table 6A) [1]. In short, during the Bachelor’s programme, students will obtain knowledge and understanding of the (physico)chemical properties of pharmaceutical ingredients and excipients, the properties of different dosage forms and basic pharmaceutical compounding.

### 6.4. Master’s Pharmacy Programme

The Pharmacy Master’s programme is designed to provide students with competencies required for professional roles in both community and hospital pharmacy in five areas of responsibility of a pharmacist: product care, patient care, medication policy, quality assurance, and research, education and innovation. Students may further specialise in the professional and clinical fields after graduation.

The learning objectives of the Pharmacy Master’s programme are described in the form of competencies belonging to the seven competency areas and in relation to these five areas of responsibilities of the pharmacist. Together, they form a matrix with 140 learning outcomes [1]. Product care is covered by multiple competency areas: while most learning outcomes can be found in the Pharmaceutical Expertise core competency, product care related learning outcomes are also part of the Communication, Knowledge and Science, and Health Advocacy and Social Responsibility competencies (Table 6B), illustrating how closely interwoven product care and patient care are in the Dutch Pharmacy programme (and in pharmacy daily practice).

### 6.5. Post-Master’s Specialisation Programmes and Post-Academic Education

After obtaining the MSc Pharmacy degree, a newly qualified pharmacist can choose to further specialise as either a community or a hospital pharmacist. The KNMP has set up the Specialists Registration Committee, which oversees the quality of these specialisations and the (re-)registration of pharmacy specialists [95].

#### 6.5.1. Community Pharmacy Specialist

The community pharmacist specialisation is a two-year programme offered by the Charlotte Jacobs Institute (CJI), the KNMP’s training institute for community pharmacy. During this post-master’s programme, the pharmacist in training is employed by a certified training pharmacy and works and learns under the supervision of an experienced community pharmacist. Following on-the-job learning, the pharmacist in training partakes in CJI-organised educational activities. Product care is an explicit part of the first year of this training programme [96].

#### 6.5.2. Hospital Pharmacy Specialist

The hospital pharmacist specialisation is a four-year programme overseen by the NVZA. Similar to the community pharmacist specialisation, the hospital pharmacist in training will learn on the job in a hospital pharmacy. The first half of the specialisation has a standard programme, which includes individual medicine preparations and reconstitution as one of the areas of expertise. In the second half, the pharmacist in training can further specialise in a specific hospital pharmacy topic by choosing a so-called differentiation (Dutch: differentiatie). Regardless of the chosen differentiation, both product care and patient care ought to be part of it [97]. Hospital pharmacists in training can further specialise by choosing the differentiation Preparations and Pharmaceutical Analysis (Dutch: Bereidingen en Farmaceutische Analyse). During this differentiation, all aspects of pharmacy preparations are prominently covered, which equips the pharmacist with broad theoretical and practical skills required for pharmacy preparations prepared in a hospital pharmacy.

#### 6.5.3. Post-Academic Education

To maintain the registration as a specialist, a pharmacist must regularly update the knowledge and skills by attending a predetermined amount of post-academic educational activities. To this end, different providers have developed courses, often in collaboration with the universities offering the Pharmacy programmes. Accreditation for these courses is regulated by the KNMP for community pharmacists and by the NVZA for hospital pharmacists [98]. Life-long learning is a must for community and hospital pharmacists.

### 6.6. Textbook

In 1992, the first edition of the textbook Recepteerkunde was published, consolidating decades of knowledge and practical experience in pharmaceutical compounding into a formalised reference. In the years that followed, Recepteerkunde served as both a guide for practicing pharmacists and a textbook for pharmacy students at Dutch universities. Five editions of Recepteerkunde were published (supported by the KNMP), gradually shifting its focus from small-scale compounding specifically to pharmaceutical product care in general.

Other European countries recognised the need for a formalised collection of knowledge and experience. At the ‘EDQM symposium on European Co-operation & Synergy and the BEAM compounding course’ in 2010, the idea for a European-wide textbook on pharmaceutical compounding was proposed. The fifth edition of Recepteerkunde would serve as the basis for this book, leading to the publication of Practical Pharmaceutics in 2015 (supported by the KNMP, NVZA and EAHP). Practical Pharmaceutics is a collaborative effort between European experts on pharmaceutical compounding and product care. It covers a wide range of topics, from basic principles (e.g., biopharmaceutics, physical chemistry and microbiology) to preconditions for compounding (such as raw materials, containers, premises and equipment) and the various specific dosage forms [99]. A second edition of Practical Pharmaceutics was published in 2023, supported by the KNMP and NVZA. This updated edition incorporates new developments and insights, such as ATMPs and 3D printing [100]. Practical Pharmaceutics has become an internationally recognised source of pharmaceutical–technological knowledge in relation to pharmacy preparations. It is used as a reference book in pharmacy practice and during the Pharmacy curriculum of universities in the Netherlands and several other countries.

## 7. Special Topics Linked to Pharmacy Preparations

This section discusses specific pharmacy preparations and related product care, primarily within hospital settings in the Netherlands. These preparations include, amongst others, customised medications tailored to individual patients and require specialised handling and care characteristics of compounding practices. Often, they are linked to research focused on product development and/or optimising pharmacotherapy with dedicated pharmaceutical formulations. The emphasis is placed on specific patient groups, product categories, and techniques employed in their preparation.

### 7.1. Optimising Individual Treatment

#### 7.1.1. Paediatric Dosage Forms

Pharmacists play a crucial role in the preparation of paediatric medications, ensuring that treatments are tailored to meet the specific needs of young patients. Major challenges include dosage adjustments, swallowability and palatability. Alternative dosage forms, such as flavoured oral solutions, suspensions, or chewable tablets, can improve adherence to treatment regimens. Age-appropriate dosage forms, such as liquid suspensions or small capsules, are prepared based on a child’s weight and age. This is particularly important for medicines that are not available in paediatric-friendly strengths [101,102,103,104].

Common excipients in medicines for adults, such as alcohol, certain preservatives, or artificial colouring agents, are less or even unsuitable for children. It is the pharmacist’s responsibility to replace these ingredients with safer alternatives or to find a way to omit them, minimising the risk of adverse effects and improving overall safety [105]. An example is the preparation of medication for paediatric patients with epilepsy who follow a ketogenic diet. The pharmacist can formulate sugar-free liquid oral solutions or capsules, ensuring that the medication is both effective and compatible with the child’s dietary restrictions.

Another important aspect of paediatric pharmacy is the tailor-made preparation of injection and infusion fluids. Although not fundamentally different from preparations for adults, this specialised type of preparation for children is often carried out by pharmacy technicians due to the increased risk of preparation errors—frequently caused by calculation mistakes—resulting from the broader dosing range required for paediatric patients. In addition, these preparations may involve more dilution steps. For example, lower doses of peptides such as insulin often require the addition of albumin as an extra formulation step to prevent the insulin from adsorption to surfaces of parts of the syringe.

Many of these preparations take place in a cleanroom located near the paediatric ward, following specialised reconstitution procedures in accordance with GMP-Z regulations.

The Netherlands maintains a national paediatric formulary (www.kinderformularium.nl (accessed on 25 May 2025)) that provides dosing guidance and lists dosage forms to achieve the calculated dose, including compounded formulations described in the FNA (Section 5.2). The formulary is well coordinated, ensuring alignment between dosing recommendations and available formulations [106]. Additionally, several FNA formulations have been explicitly designed to meet the needs of paediatric patients.

With their expertise in formulation design, drug stability, compatibility issues and paediatric pharmacology, pharmacists play an essential role in optimising paediatric care by creating safe, effective, and tailored medicines for this specific patient population.

#### 7.1.2. Swallowing Difficulties and Feeding Tubes

The correct administration of oral solid medicinal products to patients with swallowing difficulties (mostly children and the elderly) and patients with an enteral feeding tube remains a challenge. Information on the necessary adaptations is limited and associated with reduced efficacy, increased toxicity, and in the case of feeding tubes, a risk of tube obstruction.

The Oralia VTGM database (see Section 5.3) [21] provides guidance on the safe handling and transformation of solid formulations into liquid forms that can be easily swallowed or administered via a feeding tube. Instructional videos are provided as well. Whenever possible, a licensed administration route should be used as outlined in the SmPC of a product. The monographs in Oralia VTGM also suggest alternative administration routes and products to consider. In general, immediate-release tablets and unsealed (uncoated) hard capsules may undergo such handling, while sustained-release oral dosage forms may not. Unfortunately, not all immediate-release tablets can be pulverised and suspended into a viscous vehicle (suspension base, also commercially available). For these specific products, alternative approaches are provided (Table 7).

Similar information, but without technical background information, is available to healthcare providers other than pharmacy staff (e.g., nurses) through the website www.oralia.nl (accessed on 26 March 2025) [89].

For tablet crushing, commercially available manual crushers can be used, while both manual and electric grinders are available for tablet grinding. The performance of these devices varies: manual crushers generally produce a coarse powder, whereas manual or electric grinders result in a fine powder. When sufficient size reduction is required for administration via a feeding tube, as is common in hospitals and nursing homes, a grinder is preferred. To ease swallowing, oral swallowing gels are available as a substitute for water or semi-solid food for taking crushed or pulverised medication [107].

#### 7.1.3. Hospital-at-Home Care

Outpatient parenteral therapy is increasingly used in clinical practice due to its associated cost savings, reduction in inpatient hospital days, and decreased risk of hospital-acquired infections [108,109,110]. Antimicrobials were among the first types of drugs to be administered parenterally at home, a practice known as OPAT (outpatient parenteral antimicrobial therapy) [111]. To date, a wider range of parenteral drugs is administered at home, including diuretics, analgesics, immunomodulators, oncolytic agents, and TPN.

Various types of containers are used for the preparation of these medicines, such as infusion bags, cassettes, and elastomeric infusion pumps. For administration, infusion bags and cassettes require electronic infusion devices, whereas elastomeric infusion pumps are independent of a power source [112].

In the Netherlands, parenteral medication for home administration is aseptically prepared in community pharmacies, hospital pharmacies, or compounding pharmacies under GMP-Z conditions. To support the development of hospital-at-home care, several initiatives have been launched, some of which have received government funding, such as the ‘OPAT praktijkgids’ (practical guide for OPAT) [113].

### 7.2. Specialist Product Groups

#### 7.2.1. Reconstitution of Oncolytic Medicinal Products and Biologicals

Since the 1990s, it has been standard practice in the Netherlands for oncolytic medicinal products to be reconstituted in dedicated cleanrooms in hospital pharmacies rather than on the wards [114,115]. Oncolytic medicinal products include ‘classical’ chemotherapeutic agents (commonly referred to as cytotoxic medicinal products or cytotoxic drugs), oral targeted medicinal products, and biologicals.

Several factors necessitate the reconstitution of cytotoxic medicinal products in dedicated cleanrooms. Firstly, cytotoxic medicinal products often follow an individual dosing scheme, based on body surface area and at or near the maximum tolerable dose. As a result, the therapeutic window is narrow, meaning that any calculation or medication errors during reconstitution can lead to excessive toxicity or reduced efficacy. Secondly, the patient population receiving this type of medication is vulnerable and prone to myelosuppression. Strict aseptic procedures are therefore essential to prevent microbiological contamination. Lastly, cytotoxic drugs pose inherent hazards to personnel involved in their preparation. Consequently, robust safety procedures are required to prevent occupational exposure and environmental contamination.

To ensure accurate dosing, various methods are employed. When a manual procedure is used, each calculation must be verified by a second person (the ‘four-eye principle’). Additionally, validated software applications are used to calculate volumes or weights, guiding personnel through the compounding process and enabling electronic checks of in-process controls. When available, built-in cameras verify materials and drawn-up volumes. Gravimetric reconstitution, where either the syringe containing the fluid to be added to the final container is weighed, or the final container is weighed before and after addition, improves accuracy and reduces medication errors. Similarly, fully automated compounding robots (see Section 7.3.1) are employed in some Dutch hospital pharmacies, further enhancing dosing accuracy while minimising medication errors [116,117,118,119].

Over the past decades, specific safety measures have been continuously developed and improved in the Netherlands to protect staff and the environment from cytotoxic drug exposure [120,121]. The key safety principles applied during handling and reconstitution are summarised in Table 8. Longitudinal studies assessing these measures indicated that residual exposure or contamination, when present, remains below occupational safety alert levels [122]. Special attention must be given to handling vials containing commercial cytotoxic medicinal products because the majority are contaminated with trace residues of the cytotoxic drug they contain [123].

**Figure 3:**
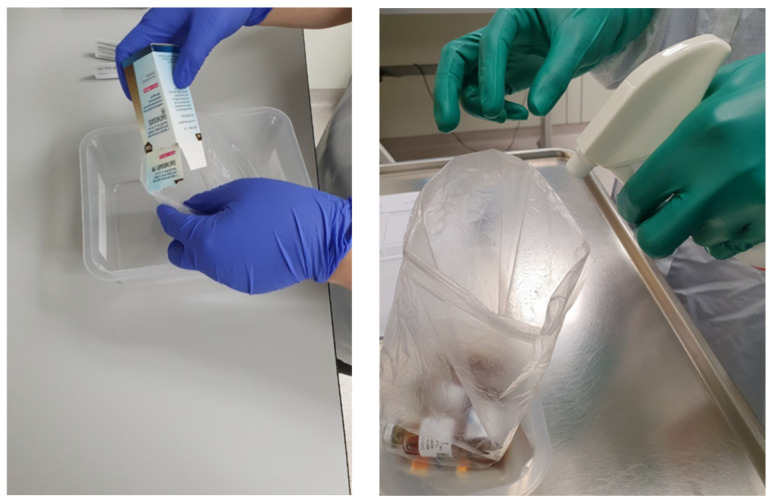
Unpacking (**left**) and disinfecting (**right**) of commercially obtained vials containing cytotoxic drugs without touching the outside of the primary packaging. Taken from [125] with permission.

Personnel involved in the reconstitution, receipt and storage of cytotoxic medicinal products must be aware of all these risks. Secondary packaging should not be removed outside the designated preparation area. Personnel responsible for unpacking and/or disinfecting the vials must be adequately trained and protected by suitable clothing. In the Netherlands, a simple, effective method has been developed for this process. First, vials are not removed from their secondary packaging by hand, but are gently slid into a small plastic bag using tweezers. Second, disinfectant is sprayed in this bag to disinfect the vials. (see Figure 3). Third, the vials are transferred from the plastic bag placed into the biological safety cabinet, again without touching them [125].

Spillage requires special attention. Clear, written procedures must be in place, along with spill kits containing the necessary materials for safe clean-up. These kits should be readily available in the compounding room, adjacent storage areas, and any rooms where ready-to-administer cytotoxic medicinal products are checked and prepared for transport. All pharmacy personnel handling cytotoxic medicinal products must receive training on spill and incident management, with mandatory annual refresher courses [124]. The appropriate response for different types of incidents involving each cytotoxic medicinal product is detailed in the national crib sheet (‘crash card oncolytics’), which is updated annually by the Working Conditions Committee of the NVZA [126].

In conclusion, the reconstitution of cytotoxic medicinal products constitutes a specific practice requiring strict precautions. The national framework of guidelines ensures the safety of both patients and healthcare workers.

#### 7.2.2. Total Parenteral Nutrition (TPN)

TPN is a vital form of nutritional support for patients who are unable to meet their oral or enteral dietary needs. It is used in both hospital and home care settings and can be tailored to adult and paediatric patients.

Due to its microbiological vulnerability, TPN is primarily prepared in hospital pharmacies under strict aseptic conditions. Any contamination poses a significant risk to patients, particularly those with compromised immune systems. In the past, TPN was often prepared as a multicomponent formulation, sometimes using computerised pump systems to automate the process [127]. To enhance the safety and reliability of TPN preparation, this approach has largely been replaced by all-in-one multicomponent infusion bags. An additional advantage of this method is cost reduction [128]. At present, most TPN preparation processes typically begin with a commercially available three-chamber bag, which contain separate compartments: for a lipid emulsion, a glucose solution and an electrolyte solution. Once these compartments are combined, the TPN can be further customised to a patient’s clinical needs by adding specific nutrients such as additional amino acids or lipids, electrolytes, vitamins, and trace elements.

To ensure compatibility and stability, pharmacists rely on information provided by the manufacturer. In some cases, TPN is entirely prepared from separate components, allowing for greater formulation flexibility. This approach is particularly useful for patients with complex nutritional needs or requiring highly individualised solutions. For example, paediatric patients with elevated liver enzymes may require customised lipid formulations to minimise hepatic stress. Similarly, patients with short bowel syndrome may need adjusted concentrations of electrolytes, additional amino acids, or fluids to compensate for altered absorption and metabolic demands [129,130,131].

Pharmacists’ specialised knowledge and expertise are essential in precisely adjusting formulations while ensuring the highest standards of safety, stability, and compatibility. They also provide support in managing technical issues, such as a clogged infusion line caused by TPN administration. In the Netherlands, specialised (hospital) pharmacies are responsible for the compounding and distribution of TPN for home care patients.

#### 7.2.3. Advanced Therapy Medicinal Products (ATMPs)

Advanced therapy medicinal products (ATMPs) have transformed the clinical landscape, demonstrating the potential to treat and even cure patients for whom conventional medicine is ineffective. According to the EMA, ATMPs are classified as either gene therapy, somatic-cell therapy, or tissue-engineered medicines. Additionally, an ATMP that includes one or more medical devices as an integral part of the medicinal product is designated as a combined ATMP. In the EU, ATMPs are considered biological medicinal products and are governed by Regulation 1394/2007 [132].

The manufacturing process of ATMPs should comply with GMP guidelines, specifically GMP Part IV—GMP Requirements for Advanced Therapy Medicinal Products [133]. Section 16 of GMP Part IV acknowledges that product reconstitution may be required after batch release, but before administration to the patient. It permits these activities to be carried out at the administration site outside a GMP-controlled environment. Examples of ATMP reconstitution include thawing, washing, centrifugation, dispersion, and dilution of the product. These handlings can take place in hospital pharmacies or dedicated facilities such as stem cell laboratories. As ATMPs may contain genetic material, viral vectors, genetically modified organisms (GMO), or cells or tissue of human origin, special cleaning and disinfection procedures may be required in the event of spillage.

Pharmacists and hospital pharmacies contribute to the research and development of ATMPs. Most Dutch academic centres have a GMP manufacturing license to produce clinical-grade ATMPs and can facilitate the translational research of in-house developed ATMPs. This also provides opportunities to conduct clinical trials for external parties. In-house manufactured ATMPs are manufactured at the hospital instead of a centralised production facility, which is typical for ATMPs with a marketing authorisation. Therefore, these products are referred to as point-of-care, place-of-care, or decentralised manufactured ATMPs.

A typical ATMP manufacturing process takes several days to weeks and consists mainly of biological processes. Compared to a conventional medicinal product, an ATMP is complex. ATMPs are inherently characterised by a considerable variability in product specifications as they are often produced using biological processes and analysed using biomolecular and biological assays. Additionally, for ATMPs manufactured from human donor material, considerable variability in the starting material is found, which may impact the final product specifications [134]. Pharmacists involved in the production, quality control testing and batch release of ATMPs should have the expertise to consider these inherent variabilities as well as the impact of deviations on the biological processes and analytical test results and, by extension, on the manufacturing process and the final drug product specification.

Pharmacists collaborate with preclinical and clinical researchers from different disciplines to facilitate the development and clinical translational research of ATMPs. During process development, pharmacists substantiate the use of non-compendial materials and processing aids. Besides supplier qualification, the materials are assessed for their suitability and the presence of impurities, mycoplasma, endotoxins, prions, and viruses by considering their origin, production processes and intended use during the ATMP manufacturing process. Furthermore, together with content experts from different disciplines, a stability study plan, risk assessments, validation protocols and reports, and batch records are compiled.

Currently, several ATMPs are being developed in Dutch academic hospitals (Table 9), with the ultimate goal of clinical application and patient access to novel therapies. To support this goal, the national consortium DARE-NL (Dutch infrastructure for cancer-specific ATMP Research) was established in 2022 and funded by the Dutch Cancer Society (KWF) [135].

#### 7.2.4. Radiopharmaceuticals and Optical Tracers

Radiopharmaceuticals are medicinal products used in nuclear medicine for diagnostic and therapeutic purposes. They consist of a tracer molecule linked to a radionuclide. The tracer molecule localises the product in the body, while the radionuclide emits radiation that scanners can detect, or delivers a radiation dose to a target area. Radionuclides decay with a specific half-life, making the handling of radiopharmaceuticals time-dependent. Nuclear imaging using positron emission tomography (PET) and single-photon emission computed tomography (SPECT) are applied for diagnostic purposes.

As their shelf life is usually limited, radiopharmaceuticals must be prepared, often requiring reconstitution, shortly before the patient undergoes a nuclear medicine procedure or treatment. In addition, dosages of prepared radiopharmaceuticals, expressed in megabecquerels (MBq), need to be customised for each patient at a specific time. As a consequence of the unique aspects of radiopharmaceuticals, compounding pharmacies play an essential role in their supply [148].

In the Netherlands, radiopharmaceuticals are prepared in a number of specialised hospital pharmacies or in centralised radiopharmacies. Hospital pharmacies supplying radiopharmaceuticals have dedicated areas designed for their preparation, which may be for either in-house use or for use in other hospitals. Alternatively, nuclear departments in Dutch hospitals can order radiopharmaceuticals from centralised radiopharmacies, which specialise in the preparation, dispensing and distribution of radiopharmaceuticals. Furthermore, due to the relatively short distances, multiple shipments take place between the main production sites and various receiving hospitals, ensuring optimal availability of radiopharmaceuticals across the country. These features make the Netherlands well suited for conducting multicentre clinical trials involving radiopharmaceuticals.

Radiopharmaceuticals are prepared in biosafety cabinets situated in a cleanroom, according to GMP-Z Annex 3 and GMP, respectively [149]. The biosafety cabinets, typically fitted with additional lead glass, protect the operators from radiation while maintaining aseptic conditions. The classification of the cleanroom is usually class C or D, depending on the outcome of a risk analysis [150].

The prescription for a radiopharmaceutical comes from a nuclear medicine physician or a nuclear radiologist. In the Netherlands, this is nearly always carried out using a nuclear medicine information system. Prescriptions specify, among others, the dosage in MBq and the time of administration. For dispensing, radiopharmaceuticals can either be ordered as ready-to-use products or prepared in-house. Ready-to-use radiopharmaceuticals only require a volume containing the correct dosage to be drawn into an injection syringe. The syringes are labelled with dose information in MBq at the time of administration and a radioactivity symbol, in addition to the general information found on labels of prepared medicinal products.

The preparation of radiopharmaceuticals often involves the reconstitution of kits. These are vials containing the tracer molecule and other necessary ingredients to prepare a radiopharmaceutical, often for multiple doses. They are mostly licensed medicinal products, with their preparation and quality control described in the SmPC. Reconstitution begins with obtaining the desired radionuclide, which is often sourced from a radiopharmaceutical generator (such as a molybdenum/technetium generator) or sometimes purchased as a precursor. The radionuclide is then added in an adjusted volume to the kit. After incubation, sometimes requiring boiling or buffer addition, the radiopharmaceutical is obtained. Quality control of reconstituted radiopharmaceuticals includes a test for radiochemical purity, for which thin-layer chromatography is mostly applied. In addition to licensed kits, some compounding pharmacies use non-licensed kits or extemporaneous preparations. For these preparations, (sterile) starting materials are procured from commercial suppliers, but can also be obtained from other compounding pharmacies [149,150].

Some Dutch hospitals (mostly university medical centres) have their own cyclotron for the production of short-lived radioisotopes, such as ^18^F, with a physical half-life of 110 min. In so-called hot cells, which are a lead-shielded GMP class C environments, radiopharmaceuticals for PET are synthesised, purified and formulated using automated synthesis modules. The final filtration step is performed in a GMP class A environment, ideally with a class B background. The preparations must comply with general and, where applicable, specific quality standards as described in the Ph. Eur. [151]. For small-scale production in the Netherlands, unlicensed diagnostic radiopharmaceuticals can, in principle, be produced in accordance with the regulations of other pharmaceutical preparations [149].

Once the preparation of the radiopharmaceutical is completed and the quality control results have been assessed, the product is released by a qualified pharmacist or a qualified person (QP). Due to the short half-life of radiopharmaceuticals, an exception is granted under GMP regulations, allowing for conditional release of the product under specific conditions before all tests (such as microbiological) are completed [152].

Following release, the radiopharmaceutical is delivered to the nuclear department in especially shielded containers to ensure safe handling. Specific transportation requirements apply to road transport, such as obtaining transport permits or notifying the Dutch nuclear safety authority [153].

Hospital pharmacists and radiopharmacists in the Netherlands are regularly consulted by nuclear medicine departments regarding preparations for special patient groups, adverse events, or drug interactions [154].

Currently, many new radiopharmaceuticals are being developed, also in (academic) hospitals, with an emerging role for theranostics. Theranostics use a single tracer molecule for both diagnostic and therapeutic purposes, enabling more precise patient selection and improved monitoring of treatment responses. Current examples include ^68^Ga-DOTA-octreotide derivatives/^177^Lu-DOTATE and ^68^Ga-PSMA or ^18^F-PSMA/^177^Lu-PSMA. For the preparation of theranostics, an increasing role for specialised compounding pharmacies is anticipated [155]. Under the new EU clinical trial regulation, GMP is no longer required for diagnostic radiopharmaceuticals used as an investigational medicinal product (IMP). However, IMP radiopharmaceuticals for therapeutic purposes must still be manufactured in accordance with GMP regulations [156].

Another recent development, with the Netherlands as frontrunner, is the use of molecular imaging in drug development and to guide treatment with targeted therapies in oncology, specifically as biomarker imaging. Monoclonal antibodies or antibody derivatives are labelled with long-lived radioisotopes, such as ^89^Zr, which matches the antibody’s biological half-life. Typical handling steps are carried out in a shielded isolator in hospital pharmacies and include radiolabelling, purification, formulation, and sterile filtration steps, immediately followed by quality control testing. An example, also used in clinical patient care, is ^89^Zr-trastuzumab for the detection of human epidermal growth factor receptor 2 (HER2)-positive tumour lesions [157]. Applications, challenges and future developments of molecular imaging in drug development and precision medicine are described in recent review papers [158,159]. In addition to labelling tracer molecules with isotopes, they can also be conjugated with optical dyes for near-infrared fluorescent molecular imaging to prepare fluorescent tracers. An advantage of this approach is the absence of radioactivity along with a generally longer shelf life, which allows for larger-scale manufacturing and stockpiling.

Fluorescent molecular imaging is complementary to nuclear imaging and provides more detailed information on the tissue and cellular level due to superior spatiotemporal resolution, whereas nuclear imaging provides the whole-body picture. Targeted tracers have been developed, based on antibodies, small molecules, peptides, nanoparticles, or proteins, and are used in various investigational applications, including image-guided surgery, pathology, endoscopy, and drug development [160]. Examples of monoclonal antibody-based optical tracers used in clinical studies are bevacizumab-800CW, cetuximab-800CW, trastuzumab-800CW, panitumumab-800CW, adalimumab-800CW, and vedolizumab-800CW [161].

The manufacturing process is performed under GMP conditions in hospital pharmacies and starts with a buffer exchange step to remove excipients, especially amino acids, from the original monoclonal antibody formulation. The dye is then coupled via direct conjugation to primary and secondary amines in the antibody, followed by removal of the free dye. The purified bulk solution is diluted in a formulation buffer and, after sterile filtration, vials are aseptically filled. After quality control, the final product is a ready-to-use solution for intravenous or topical administration [161].

A roadmap has been designed by a Dutch academic hospital for the development and clinical translation of optical tracers [162].

#### 7.2.5. Clinical Trial Medication

Extemporaneous compounding is essential for the preparation of customised formulations required for clinical trials and investigational research or for the preparation of new products used in first-in-human studies. These studies are directed to drug development and aim to compare the safety and effects of different medicines and drug delivery systems [163]. For novel drug products, suitable formulations must be developed, taking their physicochemical properties and the route of administration into account. Study medication of other drug compounds may be unavailable in the desired dose or administration form, requiring the reformulation of existing products. In phase I-II clinical trials, the final product formulation is often not yet developed. Pharmacy preparations (such as capsules or liquid formulations) enable the execution of early-phase trials while development of the final product continues.

The preparation, handling, control and documentation of clinical trial medication, designated as IMPs, are strictly regulated in the Netherlands and Europe to ensure safety, quality and efficacy. The Dutch Medicines Act applies, while on a European level, the administration of IMPs to humans is regulated by the Clinical Trials Regulation No. 536/2014, which has been effective since 31 January 2022 [156].

Although the preparation of medication for a first-in-humans study remains costly, the ability to produce innovative medicines on a small scale within a hospital or compounding pharmacy offers a unique opportunity to conduct such studies within a limited time frame and with minimal investment in production infrastructure. This approach has been effectively applied. Spray-dried tobramycin powder was formulated into a newly developed inhalation device for pulmonary delivery, Cyclops^®^, in a tolerability and pharmacokinetics study in patients with non-cystic fibrosis bronchiectasis [164]. The performance of a newly developed colon delivery system, ColoPulse^®^, was tested in healthy volunteers and in Crohn’s patients using a dual-label strategy with 13C-glucose and 15N-urea [165].

Many Dutch hospital and compounding pharmacies actively contribute to clinical studies and are generally well equipped to produce small-scale IMP batches. In addition to the examples above, their activities include the following:

- Re-labelling and encapsulation of licensed medicines for clinical trial use;
- Small-scale production of non-sterile medicines such as capsules, tablets, and dermal preparations;
- Sterile preparations, including reconstitution of trial medication produced by the pharmaceutical industry and small-scale in-house production of sterile products (e.g., biologicals, ATMPs, radiopharmaceuticals, other imaging agents, conjugated photodynamic therapy agents, and small-molecule products).

An example of support to a larger clinical study by a hospital pharmacy is the production of vitamin capsules designed for site-specific colon delivery [166]. However, for many larger clinical trial batches, production is typically carried out by specialised compounding facilities.

### 7.3. Technological Innovations

#### 7.3.1. Robotisation in Reconstitution

Since many parenteral drugs are not marketed as RTA products, several handling steps are required before each administration. When performed manually on a larger scale, this process is labour-intensive and the repetitive nature of the movements may lead to hand and joint complaints [167,168]. In addition, the need for human checks, such as the four-eye principle for verifying drawn-up fluid volumes, raises concerns regarding patient safety [49]. These factors, combined with the national shortage of personnel, have led to an increasing interest in automated and robotic reconstitution methods.

Reconstitution robots first entered the market at the beginning of the 21st century, initially focusing on the reconstitution of cytotoxic medicinal products to reduce potential personnel exposure to harmful substances [169]. The first generation of robots achieved only moderate success due to their longer production times compared to manual processes. Second- and third-generation robots have demonstrated improved productivity [170].

**Figure 4:**
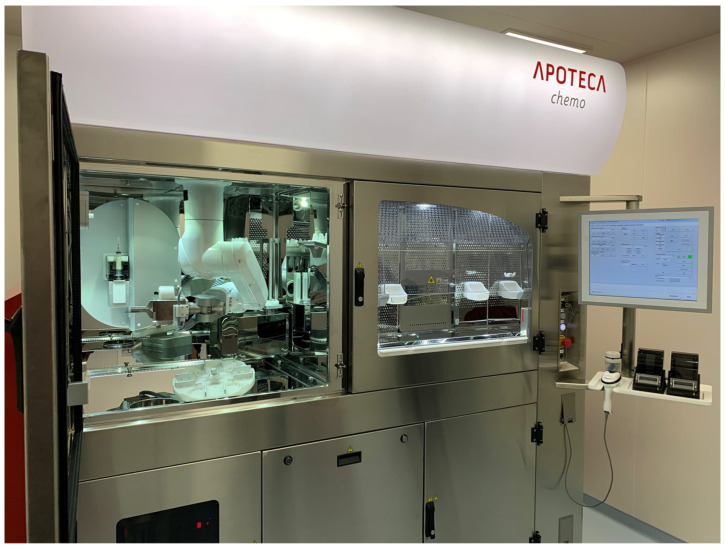
Reconstitution robot at the Department of Clinical Pharmacology and Pharmacy, Amsterdam University Medical Center, location Vrije Universiteit (photograph: MC).

As of 2024, there are four brands of reconstitution robots used in hospital pharmacies in the Netherlands: the Apoteca Chemo robot (Loccioni, Ancona, Italy), the RIVA robot (Arxium, Winnipeg, Canada), the Kiro robot (Kiro Grifols, Barcelona, Spain), and the IV Station robot (Health Robotics, Bolzano, Italy). These robots each have one or two robot arms that handle vials, bags and syringes, and feature a fully enclosed preparation area that meets GMP grade A requirements, alongside a loading and unloading area adjacent to the preparation area. The robots are capable of dissolving powders, drawing-up and adding fluids, and using gravimetric control, barcode scanning, or photo-recognition to ensure the correct medicinal product is prepared in the correct dose [167,170]. Depending on the model, the robots can either perform only individual reconstitution or a combination of individual and batch reconstitution. Additionally, some models feature automatic waste management, while others can also prepare elastomeric pumps. Figure 4 shows a reconstitution robot as operational in a Dutch hospital pharmacy.

In terms of productivity, there are no published data for RIVA or IV Station robots. Depending on the type of drug and the number of vials required per preparation, the Apoteca can compound up to 38 preparations per hour, while the Kiro can produce up to 13 preparations per hour [171,172]. Regarding dosing accuracy, robots perform as well as, or better than, manual reconstitution, with all models achieving an accuracy of 97–103% [167]. Finally, microbiological and chemical contamination performances are high, demonstrating that these robots are well suited for routine reconstitution in hospital pharmacies [173,174,175,176,177].

The number of hospital pharmacies implementing reconstitution robots is expected to increase over the coming years. Furthermore, although less advanced, several other robotic solutions are emerging to take over human tasks in compounding pharmacies. For example, visual inspection of glass ampoules for particles and the integrity of blister packaging can now be automated.

#### 7.3.2. Patient-Centric Solid Oral Dosage Forms

Currently, there is an increased demand for personalised medication and individualised therapies and a well-established lack of suitable medication with appropriate dosages and palatability for special patient groups, e.g., children. A promising approach in this context, to expand compounding capabilities, is the implementation of 3D printing technologies by which three-dimensional dosage forms are created in a layer-by-layer fashion. The size and shape of a dosage form are defined on an individual level in a computer programme that then generates a machine control code executed by a 3D printer.

Currently, different 3D printing technologies are extensively researched as manufacturing options for personalised medication. Most applicable for individual or small-scale manufacturing in pharmacies are extrusion-based technologies due to their small environmental footprint and low exposure risks. For larger-scale manufacturing, powder-based technologies such as binder jetting and selective laser sintering are used [178].

Three-dimensional printing of pharmaceutical dosage forms has multiple benefits, mainly for solid oral dosage forms. The size and shape of the dosage form can be readily modified in the computer without changing process parameters, thereby enabling personalisation of dosage and adjustment of release profiles [178]. Additionally, formulations with immediate- and sustained-release characteristics can also be developed and manufactured [179]. The technologies also enable the combination of multiple drugs in one dosage form (‘polypills’) [180,181]. Another benefit is the ability to use the same formulation irrespective of the intended dosage or shape, simplifying formulation development. This makes 3D printing a suitable technology for manufacturing clinical trial medication. Besides oral dosage forms, other solid dosage forms can be manufactured, such as suppositories [182]. Lastly, there is the possibility of incorporating commercial dosage forms into the formulation for a 3D printing process, enabling accurate individualisation of medicinal products that are still under patent protection [183].

The translation to clinical use in compounding and community pharmacies is facilitated by several companies founded in the past years providing workflows, printers, and dispensing systems.

The first hospitals and community pharmacies have acquired printing systems and are currently implementing them for use in everyday care or in clinical trials. The clinical translation of 3D printing is promising and accelerating. The first 3D printed medication as part of regular treatment will likely be provided to hospital patients in the Netherlands in 2025 and developments in community pharmacies indicate a similar timeline [184]. To ensure the highest quality standards for the future, close collaboration with the KNMP and national competent authorities is required.

Other patient-centric oral solid dosage forms that are currently receiving attention, are minitablets and orodispersible films.

With a diameter of 2–5 mm, minitablets are considerably smaller than regular-sized tablets. It has been demonstrated that minitablets are equally well accepted by neonates and children as syrup. Dose flexibility is easily achieved by administering a varying number of minitablets. As minitablets are industrially prepared for several drugs, efforts are made to develop extemporaneous compounded minitablets for a given patient, particularly in hospital settings [185].

Orodispersible films are thin films, measuring 1–2 cm by 1–2 cm, that readily dissolve or disintegrate in the mouth upon contact with saliva, after which they are easily swallowed. Three-dimensional printing of a drug substance onto a plain film appears to be the most suitable method for the extemporaneously produced, customised and patient-centred orodispersible films, although their production still has considerable limitations [186].

## 8. Conclusions, Perspectives and Challenges

Over the past decades, extemporaneous compounding has evolved from an artisan-based practice into a science grounded in knowledge and understanding. Mastery of this science is typically the domain of the pharmacist. Currently, pharmacy preparations constitute a relatively small proportion of the overall range of medicines but are indispensable to patient care. Due to their flexibility, pharmacy preparations are well suited to bridge the gap between industrially manufactured medicines and the specific needs of patients. Given the current rise in personalised medication, requiring individualised therapies based on, for example, genetic profiles, tailor-made pharmacy preparations, and the competencies to produce them, will remain a relevant and essential aspect of healthcare, providing a vital service to patients requiring tailored treatments.

New developments and perspectives have led to a resurgence of extemporaneous compounding with a renewed focus. Patient-centricity and individualised therapy are key to justifying extemporaneous compounding in (hospital) pharmacies. Additionally, patients are expected to demonstrate better adherence to tailor-made medication compared to less well-suited standard treatments, potentially leading to improved pharmacotherapeutic outcomes, reduced treatment costs, and reduced waste from unused products.

In the Netherlands, good practices include the standardisation of extemporaneously prepared medicines, continuous support from the national pharmacists’ professional association for pharmaceutical product care, and stringent and robust regulations to ensure quality and safety. Customised GMP guidelines for hospital pharmacy in the form of GMP-Z are a unique feature. The Netherlands distinguishes itself from many other countries through the continuous development of pharmacy preparations based on clinical needs. This translational research is conducted in both hospital pharmacies and centralised compounding facilities.

In addition to extemporaneous preparations, several pharmacies also manufacture stock preparations. In recent years, stock manufacturing has become increasingly centralised in specialised pharmacies, driven by both economic and quality considerations. The delivery of unique products between pharmacies is currently permitted by the IGJ and is expected to be formally incorporated into legislation in the future. The way pharmacy-to-pharmacy delivery and outsourcing are organised and implemented in the Netherlands, along with the associated quality assurance, is unique both within Europe and globally.

Future challenges will include a stronger emphasis on sustainability and green compounding, as well as integrating environmentally friendly practices into pharmacy preparations. More focus will be on the role of the pharmacist in the preparation of high-tech products, like ATMPs, vaccines and biologicals. Artificial intelligence (AI) is expected to have an impact on extemporaneous compounding. It may be a valuable tool for optimising formulations, predicting stability, easing quality control, ensuring compliance with pharmaceutical standards, and automating compounding processes. Continuous education and life-long learning are of paramount importance.

## Figures and Tables

**Table 1:** Common types of pharmacy preparations produced in compounding community pharmacies in the Netherlands.

Product Type	Specification and Examples
Topical preparations	Creams, ointments, and gels are compounded for patients who require specific formulations for skin disorders (e.g., eczema, psoriasis, or fungal infections). Lotions are used for conditions like dandruff, seborrheic dermatitis, and acne.
Oral preparations	Liquid formulations can be prepared as alternatives to medication typically available as tablet or capsule for patients who have difficulty swallowing solid medication, such as children and the elderly. Capsules can be either compounded from raw materials or from ground commercially available tablets, for example, to allow dose adaptation.
Paediatric formulations	Medicines may be compounded with flavours to make them more palatable for children. Dosages can be adjusted to suit the child’s weight or age.
Suppositories	Rectal or vaginal suppositories are compounded for medications that need to be administered via these routes, often for treating conditions such as haemorrhoids and vaginal infections.
Sterile solutions	Intravenously administered medications include antibiotic solutions dispensed in disposable infusion pumps or infusion bags for home treatment. Subcutaneous medications, such as morphine or lidocaine, are prepared for pain management when precise dosing is required or when commercial formulations are unavailable.

**Table 2:** The conditions, formulated by the IGJ, under which pharmacy-to-pharmacy delivery and outsourcing in the Netherlands are allowed [68].

Condition	Explanation
Unmet need	The compounded product may only be outsourced, compounded, and delivered if the patient’s condition cannot be adequately treated with a product that is licensed (registered) in the Netherlands. Both the dispensing and compounding pharmacies are responsible for verifying this requirement.
Notification	The compounding pharmacy is obligated to register its product in the national medicinal products database used by all healthcare stakeholders (the G-Standard, maintained and updated by Z-Index [69]). This ensures transparency regarding the products available and the pharmacy involved in pharmacy-to-pharmacy delivery.
Product file	For each product, the compounding pharmacy must maintain a file that justifies the product and its design. This file should include the medical application (pharmacotherapy), as well as the chemical and pharmaceutical aspects, such as composition, preparation method, quality controls, stability studies and expiration date, microbiological controls, and other relevant data.
GMP	The compounding pharmacy is required to comply with the EU-GMP standards, which necessitates the implementation of a fully functioning pharmaceutical quality system. Compliance is monitored by the IGJ through its on-site inspection programme. Typically, regulatory inspections are conducted every three years.
Pharmacovigilance	Compounding pharmacies must implement a pharmacovigilance system to document and assess (potential) adverse reactions on severity and causality, which should be reported to the national pharmacovigilance centre Lareb [70].
Promotion	The promotion, advertisement and/or offering of incentives for compounded products is prohibited. It is permitted to send a price list to interested dispensing pharmacies and to respond to information requests.

**Table 3:** Typical content of a monograph in Oralia VTGM [21].

Information about handling or manipulation of the medicinal product
Pharmaceutical alternatives
Therapeutic alternatives
Enteral tube administration
Enteral nutrition and food interactions
Interaction with tubing material
Route of administration
Background information on the active substance for (hospital) pharmacists
Health and safety

**Table 4:** Typical content of a monograph in Parenteralia VTGM [21].

Methods of administration
Shelf life
Influence on stability
Compatibilities
Duration/rate of administration
Maximum concentration
Other administration information
Literature

**Table 5:** Overview of all pharmacy and pharmaceutical sciences degree programmes in the Netherlands.

University	Level	Programme	Duration (ECTS) ^(1)^	Access to MSc Pharmacy? ^(2)^	Qualifies as Pharmacist?
Leiden University	Bachelor	Bio-Pharmaceutical Sciences	180	No ^(3)^	-
	Master	Bio-Pharmaceutical Sciences	120	-	No
	Master	Pharmacy	180	-	Yes
					
University of Groningen	Bachelor	Pharmacy	180	Yes ^(4)^	-
	Master	Medical Pharmaceutical Sciences	120	-	No
	Master	Molecular Medicine and Innovative Treatment	120	-	No
	Master	Pharmacy	180	-	Yes
					
Utrecht University	Bachelor	College of Pharmaceutical Sciences	180	Yes ^(5)^	-
	Bachelor	Pharmacy	180	Yes	-
	Master	Drug Innovation	120	-	No
	Master	Pharmacy	180	-	Yes
					
Vrije Universiteit Amsterdam	Bachelor	Pharmaceutical Sciences	180	No	-
	Master	Drug Discovery Sciences	120	-	No

^(1)^ In the Netherlands, 1 ECTS equals 28 h of workload. ^(2)^ Direct access, excluding additional programmes such as a pre-Master’s. ^(3)^ With the Pharmacy specialisation, this programme grants access to the MSc Pharmacy at Leiden University. ^(4)^ With the Pharmacy major. ^(5)^ With elective courses that address compounding and pharmacotherapy.

**Table 6:** Product care-related learning outcomes for the Dutch Bachelor’s (**A**) and Master’s (**B**) Pharmacy programmes [1].

**(A)**
**Bachelor’s Pharmacy Programme**
Students who complete a Bachelor of Pharmacy degree programme possess knowledge and understanding of:
The processes and factors that a play role in the route of administration and biological action of medicines and the pharmacon released in the body. The chemical and physicochemical properties and analysis of low- and high-molecular-weight active pharmaceutical ingredient and auxiliary pharmaceutical substances. The compounding of medicines in appropriate pharmaceutical dosage forms and the associated quality criteria. How the physicochemical properties of chemical compounds affect their potential use as medicine. The main patient characteristics and product properties that may influence the effects of medicines and the diagnostic measurement methods used to assess them. The processes involved in the development of medicines.
**(B)**
**Master’s Pharmacy Programme**
**Pharmaceutical Expertise**
Pharmacists are able to:
Apply a wide range of knowledge and skills to pharmaceutical matters across the full spectrum of pharmaceutical practice. ○ Apply scientific reasoning in approaching and analysing pharmaceutical matters where possible. In the area of product care this involves the application of basic principles of chemistry, physics and biology. ○ Approach and analyse pharmaceutical issues from the user’s perspective. In the case of product care, this applies primarily to the pharmaceutical dosage form and its application. Apply knowledge and skills appropriately, responsibly and ethically to relevant matters in the areas of responsibility of Product Care, Patient Care and Medication Policy in pharmaceutical practice. ○ Design high quality pharmaceutically rational and active products that are safe. ○ Assess whether a medicine meets all of the criteria to achieve the desired pharmacotherapeutic effect. ○ Select the right pharmaceutical dosage form and route of administration to achieve optimal therapeutic effect. ○ Make valid statements regarding the bioequivalence of different preparations that contain the same active substance, in the same concentration, in the same pharmaceutical dosage form. ○ Assess the rationale and feasibility of a compounding request. ○ Describe a pharmaceutical product in technical pharmaceutical and biopharmaceutical terms. ○ Develop and implement a protocol or procedure for small-batch compounding of pharmaceutical ingredients or preparation of medicines for administration. ○ Evaluate and assess the design, composition, production method and packaging of medicines. ○ Compile inspection requirements and carry out inspections. ○ Interpret the results of product inspections and make statements regarding the deliverability of products based on the interpretation of the results. ○ Determine and document optimal conditions for the transport and storage of medicines.
**Communication**
Pharmacists are able to:
Provide pharmaceutical supervision for the patient and those involved with the patient. ○ Motivate the patient and provide practical solutions in response to questions regarding, and problems with, the use of medicines or medical devices, such as difficulty with compliance. Provide verbal and written information, and report findings, regarding outcomes of (consultation on) product care, patient care, medication policy, quality assurance and their own research. ○ Keep records of relevant aspects of product care in a product file. ○ Document product care and quality assurance processes in protocols.
**Knowledge and Science**
Pharmacists are able to:
Critically assess and interpret (sources of) pharmaceutical and related medical information. ○ Develop (or contribute to the development of) protocols for patient care, medication policy, product care and quality assurance. ○ Assess the overall quality of guidelines and protocols.
**Health Advocacy and Social Responsibility**
Pharmacists are able to:
Take appropriate action in response to incidents and risks associated with product and patient care at the level of the individual patient and society. Help control pharmacotherapy costs by, among other things, monitoring and minimising spillage, dispensing in appropriate quantities, and promoting responsible recycling of unused medicines. Help protect the environment from medicine waste. ○ Show understanding of the potential environmental impact of medicines. ○ Actively promote safe disposal of unused medicines through designated facilities.

**Table 7:** Approaches in Oralia VTGM to convert an oral solid dosage form into an oral liquid dosage form. The preferred method depends on the product [21].

Disintegrate the tablet in (warm) water in a spoon, glass or cup.
Disintegrate the tablet in (warm) water in a syringe.
Crush the tablet with a tablet crusher and mix the powder with water or semi-solid food.
Open the capsule and mix the content with water or semi-solid food.

**Table 8:** Occupational safety measures for handling and reconstitution of cytotoxic medicinal products in Dutch hospital pharmacies [124].

Label all cytotoxic drugs with an adequate warning sign (a yellow hand or yellow hazard triangle).
Never touch primary packaging without wearing protective gloves.
Transport cytotoxic drugs in sealed secondary packaging.
Compound in a separate, dedicated room with entry through a lock.
Compound in a safety cabinet or isolator with negative pressure.
Do not recirculate air from the safety cabinet or isolator into the room or hospital ventilation system (0% recirculation principle).
Compound using spikes or other closed system transfer devices to limit aerosol development.
Wear adequate gloves and gowns with full length sleeves and cuffs that have been tested and proven to protect against cytotoxic substances.
Dispense compounded cytotoxic drugs with a barrier that prevents exposure of the administrating staff: a side-line or multi-way connector set filled with a neutral fluid.

**Table 9:** A selection of clinical-grade ATMPs that have been developed and investigated in clinical trials in the Netherlands.

ATMP	Indication	Institution	Manufacturing Process	Reference
Anti-CD19 CAR-T cells	Diffuse large B cell lymphoma	University Medical Center Groningen	CD4+ and CD8+ cells are enriched from leukapheresis material and ex vivo activated, transduced by a lentiviral vector, expanded, harvested, and formulated into the drug product.	[136]
Vvax-001	HPV-induced cancers	University Medical Center Groningen	RNA is produced from plasmid DNA and transfected into Vero cells that produce the Vvax-001 replicon particles, which, after purification, are formulated into the drug product.	[137]
Tumour-infiltrating lymphocytes (TIL)	Melanoma	Netherlands Cancer Institute	Melanoma lesion is surgically resected and enzymatic digested to harvest TILs, which are ex vivo expanded for 2–4 weeks, harvested, and formulated into the drug product.	[138]
Dendritic cell vaccination	Melanoma; prostate cancer	Radboud University Medical Center	Dendritic cells are isolated from apheresis material, matured, loaded with antigen, harvested, and formulated into the drug product.	[139,140]
Natural killer (NK) cells	Acute myeloid leukemia	Radboud University Medical Center	NK cells are ex vivo generated from umbilical cord blood-derived CD34+ progenitor cells using a mixture of cytokines and growth factors and are subsequently expanded, harvested, and formulated into the drug product.	[141,142]
ΔNPM1 T cell receptor (TCR)-engineered T cells	NPM1 mutated acute myeloid leukemia	Leiden University Medical Center	CD8+ cells are enriched from leukapheresis material and ex vivo activated, transduced by a lentiviral vector, expanded, harvested, and formulated into the drug product.	[143,144]
Vγ9Vδ2T cell receptor engineered T cells (TEG001)	Acute myeloid leukemia; multiple myeloma	University Medical Center Utrecht	T cells from leukapheresis material are ex vivo activated, transduced by a retroviral vector, expanded, purified, harvested, and formulated into the drug product.	[145,146]
MesoPher	Mesothelioma	Erasmus Medical Center	Monocytes are enriched from leukapheresis material and ex vivo cultured and differentiated into dendritic cells after which these dendritic cells are cultured in the presence of allogenic tumour lysate. Subsequently, the dendritic cells are matured, harvested, and formulated into the drug product.	[147]

## Data Availability

Information on some websites cited is not publicly available, but only via paid subscription or membership. Specific detailed requests can be directed to the authors and will be granted on reasonable request.

## Abbreviations

The following abbreviations are used in this manuscript:

**Abbreviation**	**Meaning**	**English Translation of Dutch** **(If Applicable)**
AI	Artificial intelligence	
API	Active pharmaceutical ingredient	
ATMP	Advanced therapy medicinal product	
BP	British Pharmacopoeia	
BSc	Bachelor of Science	
CBG	College ter Beoordeling van Geneesmiddelen	Medicines Evaluation Board (MEB)
CEP	Certificate of suitability to the monographs of the European Pharmacopoeia	
CJI	Charlotte Jacobs Institute	
DAC/NRF	Deutscher Arzneimittel-Codex/Neues Rezeptur-Formularium	German Drug Codex/New German Formulary
EAHP	European Association of Hospital Pharmacists	
ECTS	European Credit Transfer System	
EDQM	European Directorate for the Quality of Medicines and HealthCare	
EMA	European Medicines Agency	
EU	European Union	
FDA	US Food and Drug Administration	
FNA	Formularium der Nederlandse Apothekers	Formulary of Dutch Pharmacists
GCP	Good Clinical Practice	
GMO	Genetically modified organism	
GMP	Good Manufacturing Practice	-
GMP-Z	GMP-Ziekenhuisfarmacie	GMP-Hospital pharmacy
GW	Geneesmiddelenwet	Medicines Act
ICH	International Conference on Harmonisation	
IGJ	Inspectie Gezondheidszorg en Jeugd	Health and Youth Care Inspectorate
IMP	Investigational medicinal product	
KNMP	Koninklijke Nederlandse Maatschappij ter bevordering der Pharmacie	Royal Dutch Pharmacists Association
LNA	Laboratorium der Nederlandse Apothekers	Laboratory of Dutch Pharmacists
LU	Universiteit Leiden (UL)	Leiden University
MAA	Marketing Authorisation Application	
MAH	Marketing Authorisation Holder	
MIA	Manufacturing and Importation Authorisation	
MSc	Master of Science	
NVZA	Nederlandse Vereniging voor Ziekenhuisapothekers	Dutch Association of Hospital Pharmacists
OPAT	Outpatient parenteral antimicrobial therapy	
PET	Positron emission tomography	
PFSS	Prefilled sterilised syringe	
Ph. Eur.	European Pharmacopoeia	-
PIC/S	Pharmaceutical Inspection Convention/Pharmaceutical Inspection Co-operation Scheme	
PQS	Pharmaceutical quality system	
QA	Quality assurance	
QbD	Quality by design	
QP	Qualified person	
RiFaS	Risico instrument Farmaceutische Stoffen	Risk Instrument for Pharmaceutical Substances
RTA	Ready to administer	
RTU	Ready to use	
SIG	Special Interest Group	
SmPC	Summary of Product Characteristics	
SPECT	Single-photon emission computed tomography	
TPN	Total parenteral nutrition	
US(A)	United States (of America)	
USP	United States Pharmacopeia	
UG	Rijksuniversiteit Groningen (RUG)	University of Groningen
UU	Universiteit Utrecht (UU)	Utrecht University
VTGM	Voor toediening gereedmaken	Reconstitution (literally: Preparation for administration)
VU	Vrije Universiteit Amsterdam	
Wet BIG	Wet op de Beroepen in de Individuele Gezondheidszorg	Professions in Individual Health Care Act
WGBO	Wet op de Geneeskundige Behandelingsovereenkomst	Medical Treatment Contracts Act
